# Rab9A is required for delivery of cargo from recycling endosomes to melanosomes

**DOI:** 10.1111/pcmr.12434

**Published:** 2015-12-15

**Authors:** Sarmistha Mahanty, Keerthana Ravichandran, Praneeth Chitirala, Jyothi Prabha, Riddhi Atul Jani, Subba Rao gangi Setty

**Affiliations:** 1 Department of Microbiology and Cell Biology, Indian Institute of ScienceBangalore, India

**Keywords:** Rab9A, Rab38/Rab32, BLOC-1, BLOC-2, BLOC-3, AP-3, HPS

## Abstract

Melanosomes are a type of lysosome-related organelle that is commonly defective in Hermansky–Pudlak syndrome. Biogenesis of melanosomes is regulated by BLOC-1, -2, -3, or AP-1, -3 complexes, which mediate cargo transport from recycling endosomes to melanosomes. Although several Rab GTPases have been shown to regulate these trafficking steps, the precise role of Rab9A remains unknown. Here, we found that a cohort of Rab9A associates with the melanosomes and its knockdown in melanocytes results in hypopigmented melanosomes due to mistargeting of melanosomal proteins to lysosomes. In addition, the Rab9A-depletion phenotype resembles Rab38/32-inactivated or BLOC-3-deficient melanocytes, suggesting that Rab9A works in line with BLOC-3 and Rab38/32 during melanosome cargo transport. Furthermore, silencing of Rab9A, Rab38/32 or its effector VARP, or BLOC-3-deficiency in melanocytes decreased the length of STX13-positive recycling endosomal tubules and targeted the SNARE to lysosomes. This result indicates a defect in directing recycling endosomal tubules to melanosomes. Thus, Rab9A and its co-regulatory GTPases control STX13-mediated cargo delivery to maturing melanosomes.

**Significance**

Rab GTPases regulate cargo transport by controlling membrane fusion between organelles. One of 60 known Rabs, Rab9A, has been shown to function in recycling cargo from late endosomes to the Golgi in non-melanocytes. Our analyses showed that Rab9A is required for melanocyte pigmentation and cargo transport. This is similar to the requirement for Rab38/32 or their GEF BLOC-3 complex, indicating that Rab9A may function in the same trafficking pathway to melanosomes. In addition, Rab9A, Rab38/32 and BLOC-3 regulate the length of recycling endosomes and target the fusion SNARE-STX13 to lysosomes upon their knockdown, suggesting a role for these molecules in stabilizing the fusion of recycling endosomal tubules with melanosomes.

## Introduction

The endosomal system in mammalian cells is extremely dynamic and generates several structurally and functionally distinct compartments, namely early/recycling endosomes, late endosomes, and lysosomes. In addition, certain cell types produce specialized compartments with unique functions, collectively known as ‘lysosome-related organelles’ (LROs) (Dell’Angelica et al., 2000). Melanosomes are one such LRO and they utilize recycling endosomes for their biosynthesis in melanocytes (Raposo et al., 2007). Defects in the function or formation of melanosomes result in oculocutaneous albinism, a clinical phenotype commonly observed in Hermansky–Pudlak syndrome (HPS). HPS is caused by mutations in any of nine human genes or their orthologs, and six other genes in mouse (Huizing et al., 2008; Wei, 2006). The protein products of these genes have been grouped into several multisubunit cytosolic protein complexes, including BLOC (biogenesis of lysosome-related organelles complex) -1, -2, -3, adaptor protein (AP)-3 and HOPS (homotypic vacuolar protein sorting) (Raposo and Marks, 2007). However, the roles of these complexes in melanosome biogenesis are only partially understood (Marks et al., 2013; Sitaram and Marks, 2012).

Melanosome biogenesis initiates at vacuolar early endosomes (stage I), which segregate into non-pigmented premelanosomes (stage II) characterized by the presence of PMEL (premelanosomal protein) fibrils (Raposo et al., 2001). These organelles become pigmented due to the transport of melanin-synthesizing enzymes TYRP1 (tyrosinase-related protein-1), TYR (tyrosinase) and other proteins from tubular/vesicular recycling endosomes and mature into stages III and IV (Marks et al., 2013; Sitaram and Marks, 2012). Previous studies have shown that mutations in the subunits of BLOC-1, -2, -3 or AP-3 block the maturation of melanosomes from stage II to stage III/IV, suggesting a defect in melanosomal cargo transport from recycling endosomes (Dennis et al., 2015; Di Pietro et al., 2006; Gerondopoulos et al., 2012; Salazar et al., 2006; Setty et al., 2007; Sitaram et al., 2012; Theos et al., 2005). In addition, BLOC-1-deficient melanocytes accumulate TYRP1, ATP7A, and other cargo in enlarged vacuolar early endosomes. This accumulation is followed by continuous recycling and internalization of the cargo molecules through the cell surface (Setty et al., 2007, 2008), indicating that BLOC-1 functions in either the generation of tubular recycling endosomal structures or extracting cargo into tubular structures for melanosome maturation. Furthermore, the adaptor AP-1 and its interacting kinesin motor protein, KIF13A, are required for the formation of recycling endosomal tubular structures (Delevoye et al., 2009), while the BLOC-2 complex is required for targeting these tubular intermediates to the melanosomes (Dennis et al., 2015). In contrast, AP-3 was shown to sort TYR through a dileucine motif (Honing et al., 1998) on a different endosomal domain, which mediates TYR transport to melanosomes (Chapuy et al., 2008; Huizing et al., 2001; Theos et al., 2005). These studies have established the existence of two independent pathways: (i) transport of TYRP1 and other cargo by BLOC-1, -2 (referred to here as BLOC-1-dependent) and (ii) trafficking of TYR by AP-3 (referred to here as AP-3-dependent) from recycling endosomes to maturing melanosomes (Marks et al., 2013; Sitaram and Marks, 2012). In addition, STX13 (Prekeris et al., 1998), a BLOC-1-interacting recycling endosomal SNARE (Ghiani et al., 2010; Moriyama and Bonifacino, 2002), and the actin nucleator WASH (Wiskott–Aldrich Syndrome Protein and SCAR Homolog) complex (Ryder et al., 2013), have also been implicated in melanosome biogenesis.

Rab GTPases cycle between the cytosol and distinct membrane domains, and they recruit sets of specific effector proteins that act in concert to mediate membrane fusion, receptor segregation, cargo packaging or organelle motility (Pfeffer, 2013; Stenmark, 2009). In addition, Rabs interact with tethering proteins to facilitate SNARE-mediated membrane fusion events (Kummel and Ungermann, 2014). In melanocytes, several Rabs and their effectors, including SNAREs, have been shown to regulate cargo transport to melanosomes. The depletion of Rab7 (Hida et al., 2011), VARP (VPS9-ankyrin-repeat protein, an effector protein of Rab38/32) (Tamura et al., 2009), or VAMP7 (vesicle-associated membrane protein 7, a binding partner of VARP) (Tamura et al., 2011) misroute the BLOC-1-dependent TYRP1 cargo and cause melanocyte hypopigmentation. However, the role of these proteins in TYR (AP-3-dependent) transport has not been addressed. Furthermore, siRNA-mediated inactivation of either Rab38 or 32, or genetic mutation of *RAB38* in mouse has been shown to mistarget both TYRP1 and TYR, resulting in hypopigmented melanosomes (Bultema et al., 2012; Wasmeier et al., 2006). Moreover, BLOC-3 is known to function as a GEF (guanine exchange factor) for Rab38/32, and its deficiency causes hypopigmentation similar to that observed in Rab38/32 knockdown melanocytes (Gerondopoulos et al., 2012). Interestingly, the HPS4 subunit of BLOC-3 was shown to interact with endolysosomal Rab9A in non-melanocytes (Kloer et al., 2010), but its precise role in melanosome biogenesis remains unknown. Studies have suggested that Rab9A acts either upstream of or in concert with BLOC-3 or Rab38/32 proteins in the transport steps to melanosomes (Marks, 2012). However, none of these studies clearly demonstrated the function of Rab9A in transport steps to melanosomes.

In this study, we sought to understand the role of Rab9A in cargo trafficking from recycling endosomes to melanosomes. We used immunofluorescence and live cell microscopy to study the cargo localization or dynamics of tubular recycling endosomal structures in melanocytes obtained either from HPS models or through shRNA-mediated gene knockdown. Our goal was to place Rab9A in the BLOC-3-Rab38/32-VARP (referred to here as Rab9A co-regulators) pathway that functions linearly in the biogenesis of LROs such as melanosomes. Our data demonstrate that Rab9A regulates the trafficking of both BLOC-1- and AP-3-dependent cargo by regulating recycling endosomal fusion events with melanosomes, similar to the regulation carried out by BLOC-3, Rab38/32, and VARP.

## Results

### Rab9A localizes to lysosomes and associates with melanosomes in wild-type melanocytes

**Figure 1:**
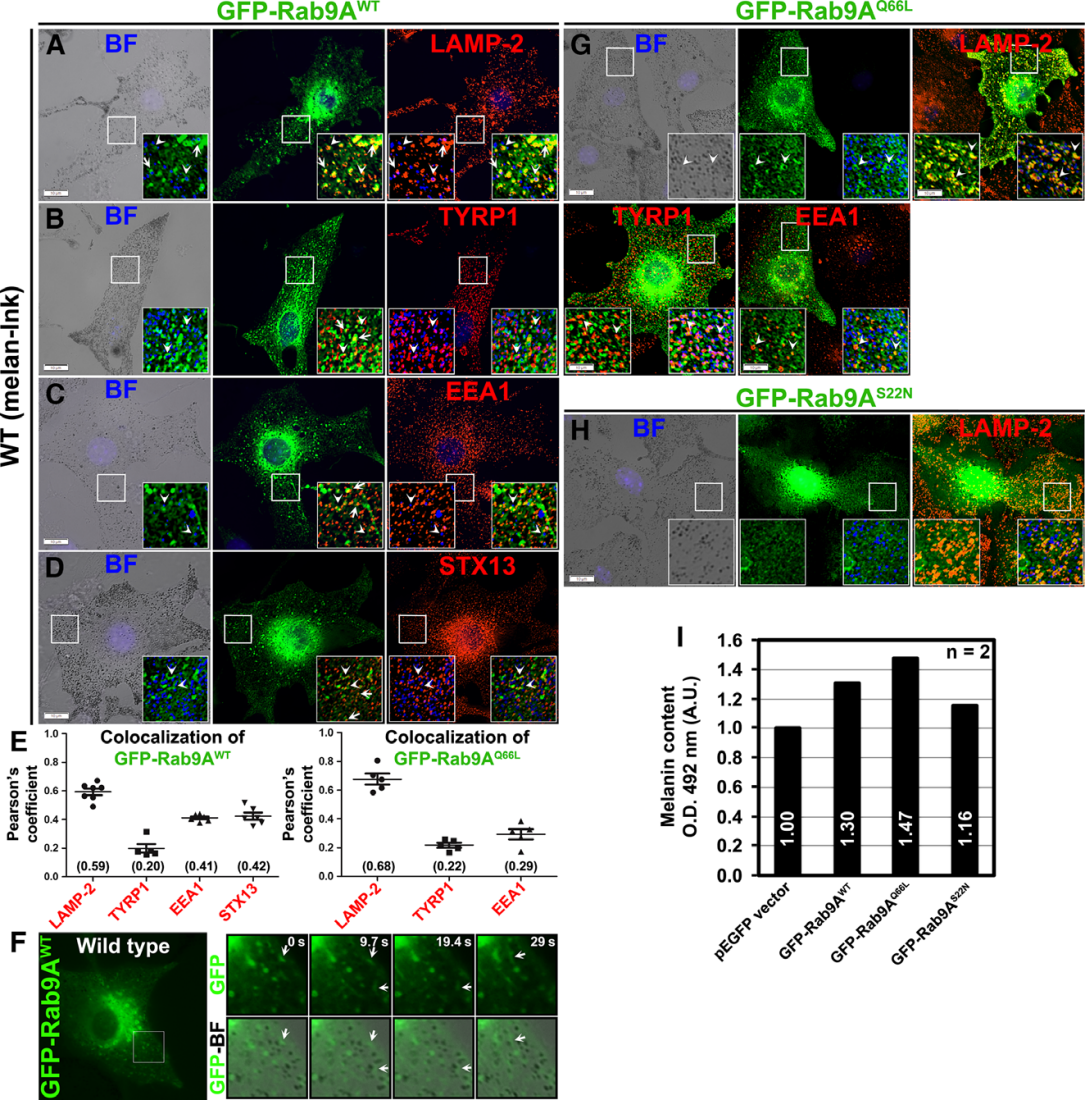
Localization and function of Rab9A in wild-type melanocytes. (A–E, G) Rab9A^WT^ and Rab9A^Q^^66L^ localizes primarily to lysosomes, with a subset in melanosomes and endosomes. Wild-type melanocytes were transiently transfected with 1 μg of GFP-Rab9A^WT^ or GFP-Rab9A^Q^^66L^ and then analyzed by BF and IFM. Arrows point to tubular GFP-Rab9A structures that are colocalized or associated with LAMP-2 (A) or EEA1 (C) or STX13 (D), in proximity to TYPR1 (B) or associated with BF melanosomes (pseudocoloured to blue) (A-D). Arrowheads point to the ring-like or punctate GFP-Rab9A (WT or Q66L) structures that are colocalized with LAMP-2 and melanosomes (pseudocoloured to blue) (A, G), TYRP1 (B, G), EEA1 (C, G), or STX13 (D). Graph (E) represents the colocalization efficiency between GFP-Rab9A (WT or Q66L) and other markers measured as Pearson’s correlation coefficient (*r*, n = ∼5–7 cells). Values in parenthesis indicate the colocalization coefficient between the two marker proteins. (F) Tubular GFP-Rab9A^WT^ structures associated with melanosomes in live imaging microscopy. Cells were transfected with 1 μg of GFP-Rab9A and then imaged by live imaging microscopy. Insets represent GFP-Rab9A localization alone (top panel) or relative to melanosomes (bottom panel) at different time points. Arrows point to the tubular GFP-Rab9A structures. (H) Rab9A^S^^22N^ localizes to cytosol in melanocytes. Wild-type melanocytes were transiently transfected with 1 μg of GFP-Rab9A^S^^22N^ and then analyzed by BF and IFM. (I) Overexpression of Rab9A increases the melanocyte pigmentation. Wild-type melanocytes were transiently transfected with 2 μg of different isoform of GFP-Rab9A or empty vector (as a control) and then measured the melanin pigments (n* *=* *2). Values indicate the fold change in pigmentation relative to control.

Several Rab GTPases, such as Rab7, 27, 32, 38, have been shown to function in melanosome biogenesis or organelle motility (Ohbayashi and Fukuda, 2012). Moreover, Rab38 and 32 regulate the transport steps to melanosomes in human melanocytes by localizing to the limiting membrane of melanosomes or early endosomes (Bultema et al., 2012; Gerondopoulos et al., 2012). We examined whether Rab9A localizes to melanosomes and regulates their biogenesis in mouse melanocytes. However, Rab9A in non-melanocytes localizes primarily to late endosomes and functions in retrograde transport from either endosomes (Chia et al., 2011) or late endosomes (Lombardi et al., 1993) to Golgi. Here, immunofluorescence microscopy (IFM) of wild-type (melan-Ink4a) melanocytes showed that GFP-Rab9A^WT^ (referred to here as GFP-Rab9A) localized to tubular and punctate structures that were in contact or colocalized with LAMP-2, a protein enriched in lysosomes (Figure1A, *r* = 0.59 ± 0.02 in 1E left panel). Interestingly, few of these Rab9A-positive structures were closely associated with pigmented melanosomes (pseudocoloured blue, arrows in Figure1A–D). Consistently, a cohort of tubular Rab9A structures were in close proximity to or contact with the melanosome protein TYRP1 (arrows and arrowheads in Figure1B, *r* = 0.2 ± 0.03 in 1E left panel), suggesting that Rab9A is associated with melanosomes. Although Rab9A was previously shown to localize to late endosomes in kidney cells (Lombardi et al., 1993), we found a cohort of Rab9A localized to EEA1-positive early endosomes in melanocytes (arrowheads in Figure1C, *r* = 0.41 ± 0.01 in 1E left panel). Similarly, a subset of tubular Rab9A structures were associated or colocalized with STX13-positive recycling endosomes (arrows and arrowheads in Figure1D, *r* = 0.42 ± 0.03 in 1E left panel). However, few of the Rab9A-positive melanosomes were also labeled for LAMP-2 (arrowheads in Figure1A). Furthermore, GFP-Rab9A in wild-type melanocytes appeared as enlarged ringlike structures in live cell microscopy, and a small number of Rab9A-positive tubular structures were associated with melanosomes (arrows, Figure1F and Video S1). In addition, Rab9 co-fractionated with melanosome (TYRP1, TYR or partly with Rab38) and lysosome (LAMP-2) proteins and a cohort of Rab9 was observed in the endosomal fractions (positive for Rab4 or EEA1) (Figure S1A). Consistently, expression of constitutively active isoform of Rab9A (Rab9A^Q66L^) in wild-type melanocytes showed a slight increase in Rab9A localization to both lysosomes and melanosomes, but not to the EEA1-positive endosomes (arrowheads in Figure1G; *r *=* *0.68 ± 0.04 for lysosomes, 0.22 ± 0.02 for melanosomes and 0.29 ± 0.04 for endosomes in 1E right panel). As expected, dominant-negative isoform of Rab9A (Rab9A^S22N^) localizes to cytosol in wild-type melanocytes (Figure1H). Accordingly, the expression of either Rab9A WT or Q66L substantially increased the melanocyte pigmentation compared to Rab9A S22N isoform or empty vector (Figure1I). Moreover, Rab9A and its isoform expression in wild-type melanocytes slightly increased the protein stability of both melanosome and lysosome cargoes but not the transcripts expression (Figure S1B, C). These results indicate that Rab9A localizes primarily to lysosomes and also partially associates with melanosomes and early/recycling endosomes, suggesting a possible role in melanosome biogenesis and transport.

### Rab9A requires Rab38 for melanocyte pigmentation

**Figure 2:**
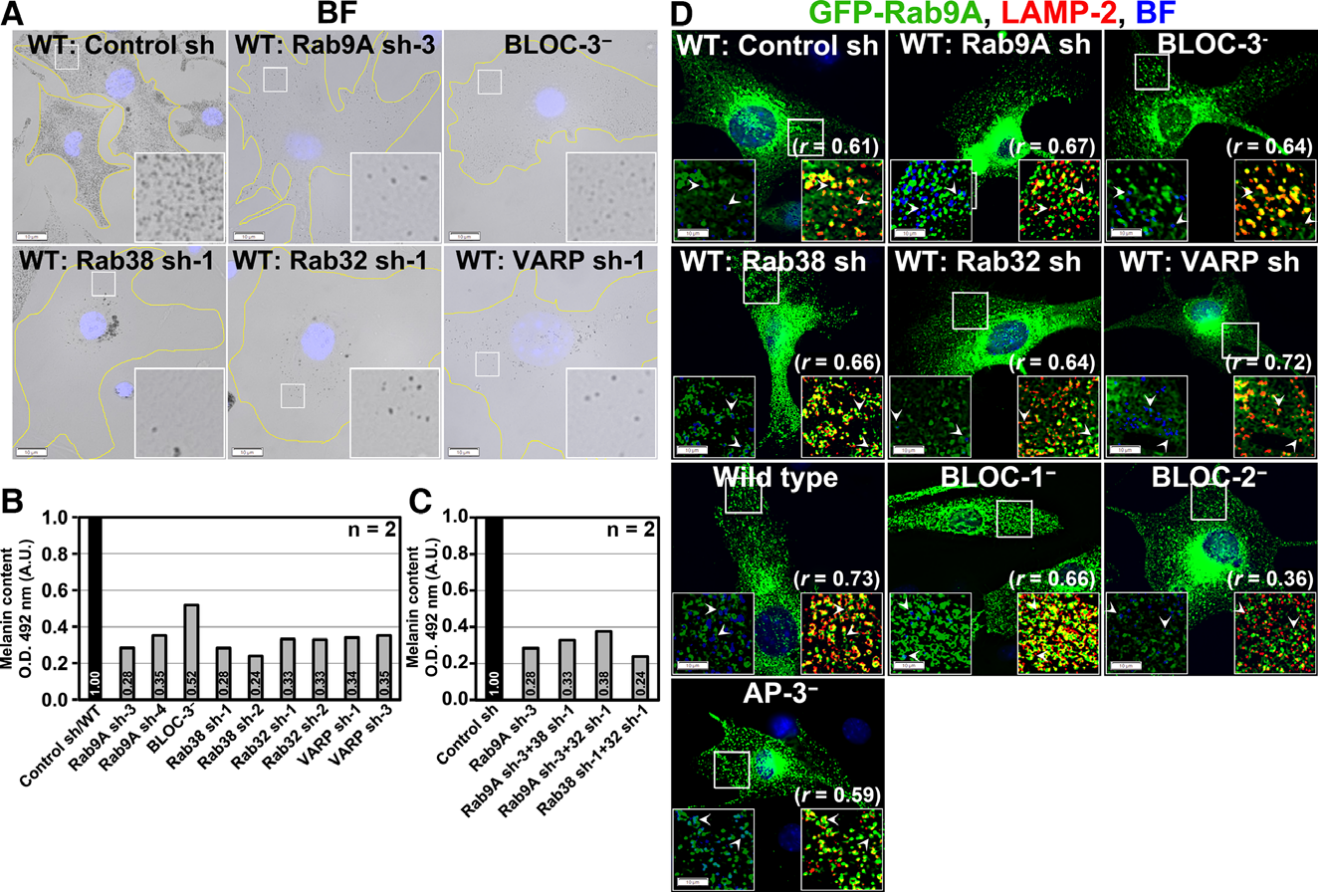
ShRNA-mediated depletion of Rab9A in wild-type melanocytes. (A) Rab9A knockdown results in hypopigmentation, similar to BLOC-3^−^ cells or Rab38/32 or VARP-depleted melanocytes. Cells were transduced with retrovirus encoding shRNA against *RAB9A*, *RAB38*, *RAB32,* or *VARP* genes and analyzed by BFM. (B and C) Rab9A depletion affects the melanocyte pigmentation, similar to Rab38/32 or VARP-knockdown or Rab9A-38 or Rab9A-32 double knockdown. Wild-type melanocytes were transduced with retrovirus encoding respective shRNA as labeled and measured the melanin pigments (n* *=* *2). BLOC-3^−^ cells are mouse melanocytes deficient for HPS4 subunit. Values indicate the fold change in pigmentation relative to control. (D) Localization of GFP-Rab9A to lysosomes increases upon knockdown of Rab38/32 or VARP, or in BLOC-1^−^, −3^−^, or AP-3^−^ melanocytes. Cells were transiently transfected with 2 μg of GFP-Rab9A and then analyzed by BF and IFM. Arrowheads point to tubular or ring-like GFP-Rab9A structures that are positive for LAMP-2 in all cells and closely associated or colocalized with hypopigmented melanosomes (pseudocoloured to blue) in knockdown cells. Note that GFP-Rab9A localization to LAMP-2-positive organelles is significantly decreased in BLOC-2^−^ cells. Nuclei were stained with Hoechst. Bars, 10 μm and insets, 2.5X of white boxed regions.

We examined whether Rab9A regulates melanocyte pigmentation. Our semi-quantitative PCR analysis of Rab9 showed wild-type (melan-Ink4a) melanocytes express higher transcript levels of *RAB9A* compared to its paralog *RAB9B* (0.6 and 0.3 times of *GAPDH*, respectively) (Figure S2A). Compared to control cells, melanocytes transduced with retrovirus encoding three different shRNAs against *RAB9A* displayed hypopigmentation of ∼75–80% of melanocytes (Figures2A and S2F), consistent with reduced transcript levels of *RAB9A* but not *RAB9B* and the total Rab9 protein in these cells (Figure S2B,C). Previous studies have suggested that Rab9A functions upstream of BLOC-3 and Rab38/32 in melanosomal transport (Gerondopoulos et al., 2012; Kloer et al., 2010). Moreover, it has been shown that Rab38/32 interacts with VARP and BLOC-2 (Bultema et al., 2012; Tamura et al., 2009), while BLOC-1 interacts with BLOC-2 and AP-3 independently (Di Pietro et al., 2006). To test whether Rab9A functions in a manner similar to Rab38/32, BLOC-3, VARP, or BLOC-1, -2, AP-3 in melanosome cargo transport, we generated specific shRNAs against *mRAB38*, *mRAB32,* and *mVARP* in a retroviral vector and transduced them into wild-type melanocytes. It has been shown that the expression level of Rab38 and 32 is dependent on cell type (Wasmeier et al., 2006). Our semi-quantitative PCR analysis of Rab38 and 32 transcripts showed almost equal expression of the two (2.7 and 2.3 times of *GAPDH*, respectively) in melan-Ink4a wild-type mouse melanocytes (Figure S2A). Knockdown efficiency of Rab38, 32, or VARP in melanocytes was confirmed by semi-quantitative transcript analysis (Figure S2D,E) and was consistent with the loss of pigmentation in ∼65–75% of melanocytes compared to cells transduced with control shRNA (Figure S2F). In addition to these cells, we also used immortal melanocytes derived from BLOC-3 (melan-le, BLOC-3^−^), BLOC-2 (melan-coa, BLOC-2^−^), BLOC-1 (melan-mu, BLOC-1^−^), and AP-3 (melan-mh, AP-3^−^)-deficient HPS mouse models (Figures2A and S2G). Next, we compared the pigmentation of Rab9A-depleted cells with Rab38, 32, and VARP knockdown melanocytes or BLOC-1, 2, 3-deficient melanocytes. Notably, bright field microscopic (BFM) analysis revealed that hypopigmentation in Rab9A-depleted melanocytes was similar to that observed in the melanocytes knocked down for Rab38, Rab32, or VARP, as well as in mutant BLOC-3 cells (Figure2A), consistent with the reduced melanin pigments observed in these cells compared to control or wild-type melanocytes (Figure2B). Furthermore, pairwise knockdown of Rab9A and Rab32 or Rab38 had no substantial change in melanocyte pigmentation compared to the individual gene depleted cells (Figure2C). Moreover, the hypopigmentation phenotype observed in Rab9A-knockdown cells was also comparable to that observed in the BLOC-2 or AP-3-deficient melanocytes (Figure S2G). These results indicate that the depletion of Rab9A affects pigmentation in melanocytes.

**Figure 3:**
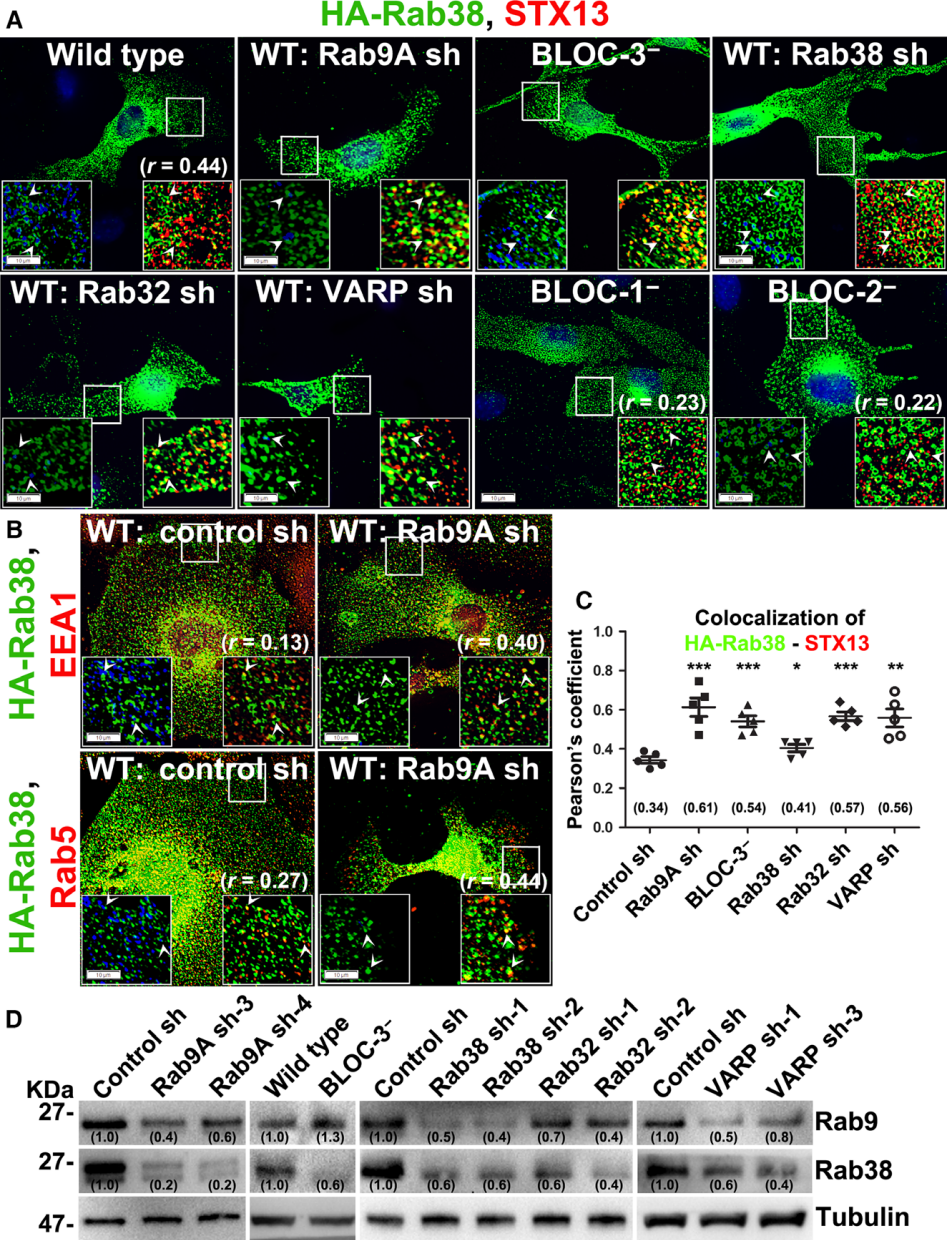
Localization and steady-state levels of Rab38 in Rab9A-knockdown cells. (A, C) HA-Rab38 localizes to melanosomes in wild type and BLOC-2-deficient cells. Its localization to recycling endosomes increases upon BLOC-3-deficiency or knockdown of Rab9A, Rab32 or VARP in wild-type melanocytes. Cells were transiently transfected with 2 μg of HA-Rab38 and analyzed by BF and IFM. Arrowheads point to the Rab38 localization with respect to melanosomes (pseudocoloured to blue) or endosomal STX13. Graph (C) represents the colocalization efficiency between HA-Rab38 and STX13 measured as Pearson’s correlation coefficient (*r*, n* *=* *5 cells). Values in parenthesis indicate the colocalization coefficient between HA-Rab38 and STX13. &, P < 0.05; &&, P < 0.01 and &&&, P < 0.001; mean±s.e.m. (B) Localization of HA-Rab38 to early endosomes increases upon Rab9A-depletion in wild-type melanocytes. Rab9A-knockdown or control cells were transfected with HA-Rab38 and analyzed by BF and IFM. Arrowheads point to the Rab38 localization with respect to melanosomes (pseudocoloured to blue) or endosomal EEA1 or Rab5. Values in parenthesis indicate the colocalization coefficient between HA-Rab38 and EEA1 or Rab5. Nuclei were stained with Hoechst. Bars, 10 μm and insets, 2.5X of white boxed regions. (D) Rab9 expression is slightly but Rab38 expression significantly reduced in Rab9A, Rab38/32 or VARP-knockdown or BLOC-3^−^ melanocytes. Immunoblot analysis of cellular Rab9 and Rab38 protein levels, with *γ*-Tubulin as a loading control. Values in parenthesis indicate the fold change in band intensity relative to control. Note that the band intensities were normalized with their respective tubulin prior to the fold change calculation.

To test whether Rab9A works in concert with Rab38/32, we overexpressed Rab9A in Rab38 or 32-knockdown melanocytes and analyzed the pigmentation by BFM. We hypothesized that if Rab9A functions independent of Rab38/32; its overexpression should rescue the hypopigmentation of Rab38/32-deficient melanocytes. Ectopic expression of GFP-Rab9A in Rab38 or 32-knockdown melanocytes, as well as VARP-depleted melanocytes, had no effect on cellular hypopigmentation. In addition, pigmentation was not altered in BLOC-1, -2, and AP-3-deficient melanocytes upon Rab9A overexpression, suggesting that Rab9A alone is not sufficient to rescue the pigmentation of these cells (Figure S2H). Consistent with these results, GFP-Rab9A (shRNA-sensitive human Rab9A) partially rescued the pigmentation phenotype of Rab9A-knockdown melanocytes (Figure S2H). Importantly, IFM studies showed that gross localization of Rab9A to lysosomes (positive for LAMP-2) was unaffected in melanocytes deficient for Rab38, Rab32, VARP, BLOC-1, or the AP-3 complex (Figure2D; *r *=* *0.61 ± 0.02 in control sh, 0.67 ± 0.02 in Rab9A sh, 0.64 ± 0.01 in BLOC-3^−^, 0.66 ± 0.03 in Rab38 sh, 0.64 ± 0.02 in Rab32 sh, 0.72 ± 0.02 in VARP sh, 0.73 ± 0.01 in wild type, 0.66 ± 0.03 in BLOC-1^−,^ and 0.59 ± 0.01 in AP-3^−^). Interestingly, Rab9A localization to LAMP-2-positive organelles was significantly reduced in BLOC-2-deficient melanocytes (Figure2D; *r *=* *0.36 ± 0.02), which might be due to altered LAMP-2 trafficking in these cells (see below) (Falcon-Perez et al., 2005). These results suggest that lysosomal localization of Rab9A is independent of Rab38/32, VARP, BLOC-1, and AP-3 molecules. Likewise, Rab9A-positive tubular structures associated with hypopigmented melanosomes were unchanged in Rab38-, 32- or VARP-inactivated melanocytes (arrows in Figure2D). In contrast, colocalization of Rab9A with hypopigmented melanosomes increased in BLOC-1^–^, −2^–,^ and AP-3^–^ compared to wild-type cells (Figure2D). However, the endogenous levels of Rab9A but not Rab4 (endosomal Rab used as a control) were slightly reduced, similar to the decreased number of hypopigmented melanosomes observed in the Rab38, Rab32, VARP-deficient, or Rab38-32 double-deficient melanocytes (see Figures3D and S3C). Together, these results indicate that partial localization or association of Rab9A to melanosomes in wild-type melanocytes is independent of these regulatory proteins.

We tested whether hypopigmentation of Rab9A-depleted cells could be rescued with the overexpression of Rab38, a key GTPase shown by several studies to be required for melanosome biogenesis (Bultema et al., 2012; Gerondopoulos et al., 2012; Wasmeier et al., 2006). Expression of HA-Rab38 in melanocytes depleted for Rab9A or other regulatory molecules did not compensate the pigmentation loss, suggesting that Rab38 expression alone is not sufficient to rescue the pigmentation (Figure S2I). As expected, HA-Rab38 (human, shRNA susceptible) partially rescued the pigmentation of Rab38-knockdown melanocytes (Figure S2I). In melanocytes, Rab38 has been shown to localize to the melanosomes (Bultema et al., 2012; Wasmeier et al., 2006). Therefore, we tested whether this localization is dependent on Rab9A or any other regulators. IFM studies showed HA-Rab38 in wild-type melanocytes predominantly localized to pigment granules, while a subset was colocalized with STX13-positive recycling endosomes (*r *=* *0.44 ± 0.03) (Figure3A). HA-Rab38 localization to the melanosomes was substantially reduced in melanocytes deficient for Rab9A, BLOC-3, Rab32, and VARP, but not in BLOC-2^–^ cells (Figure3A). This is possibly due to lack of mature melanosomes in these cells (Figure3A). Correspondingly, localization of HA-Rab38 to STX13-positive endosomes significantly increased in Rab9A, 32, VARP-knockdown, and BLOC-3-deficient melanocytes (Figure3A,C; *r *=* *0.61 ± 0.05 in Rab9A sh, 0.54 ± 0.03 in BLOC-3^−^, 0.57 ± 0.02 in Rab32 sh and 0.56 ± 0.05 in VARP sh compared to 0.34 ± 0.02 in control sh). Consistently, HA-Rab38 localization to EEA1-positive (*r *=* *0.40 ± 0.03 in Rab9A sh and 0.13 ± 0.01 in control sh) or Rab5-positive (*r *=* *0.44 ± 0.02 in Rab9A sh and 0.27 ± 0.01 in control sh) endosomes moderately increased in Rab9A-knockdown compared to control melanocytes (Figure3B). Moreover, HA-Rab38 in BLOC-1-deficient melanocytes appeared as punctate and enlarged ring-like structures that partially colocalized with STX13 (Figure3A, *r* = 0.23 ± 0.04). As expected, HA-Rab38 localization to melanosomes was partially restored and its colocalization with STX13 modestly reduced (*r *=* *0.41 ± 0.02) in Rab38-depleted melanocytes (Figure3A). In addition, endogenous levels of Rab38 were reduced in Rab9A, Rab32, VARP-knockdown, and BLOC-3^−^ melanocytes (Figure3D). These results suggest that Rab38 localizes primarily to endosomes in the absence of mature melanosomes, and its recruitment to the melanosomes is dependent on Rab9A, Rab32, and VARP. Together, these results indicate that Rab9A and Rab38 are required for the biogenesis of melanosomes.

### Rab9A regulates cargo transport to melanosomes

**Figure 4:**
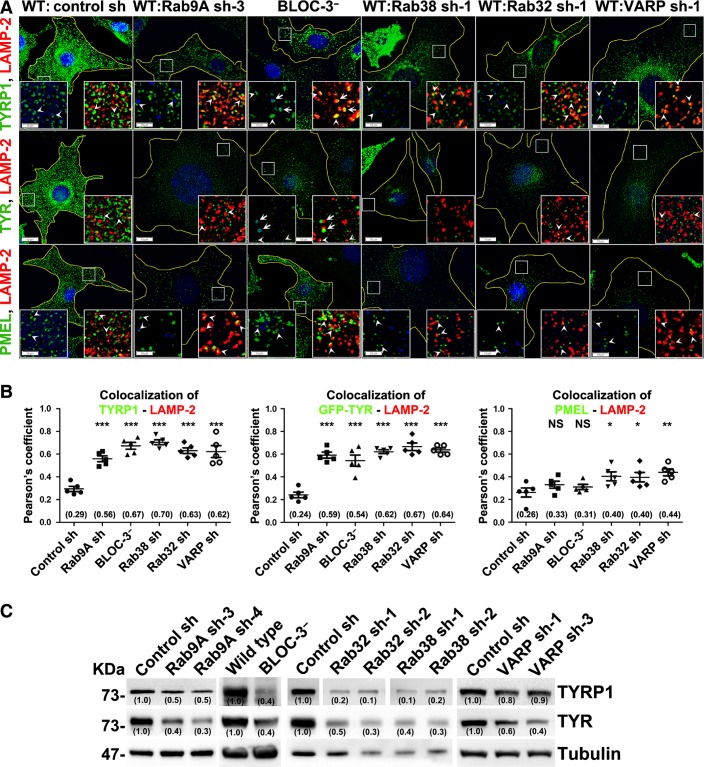
Steady-state distribution of melanosomal proteins and their expression in Rab9A-depleted melanocytes. (A, B) Rab9A knockdown leads to mislocalization of melanosome cargo to lysosomes, similar to the phenotype observed in BLOC-3^−^, or Rab38/32, or VARP-knockdown melanocytes. Cells were fixed, stained for melanosomal proteins and analyzed by BF and IFM. Arrowheads point to the melanosomal proteins TYRP1, TYR, and PMEL and their localization relative to either the lysosomal protein LAMP-2 or melanosomes (pseudocoloured to blue). Arrows point to the localization of cargo to melanosomes that are colocalized or associated with LAMP-2 in BLOC-3-deficient melanocytes. Nuclei were stained with Hoechst. Bars, 10 μm and insets, 2.5X of white boxed regions. Graph (B) represents the colocalization efficiency between TYRP1 or GFP-TYR or PMEL and LAMP2 measured as Pearson’s correlation coefficient (*r*, n* *=* *5 cells). Values in parenthesis indicate the colocalization coefficient between the two marker proteins. ns, not significant; &, P < 0.05; &&, P < 0.01 and &&&, P < 0.001; mean ± SEM. (C) Melanosomal protein expression is dramatically reduced in Rab9A, Rab38/32 or VARP-knockdown or BLOC-3-deficient melanocytes. Immunoblot analysis of melanosomal proteins TYRP1 and TYR, with *γ*-Tubulin as a loading control. Values in parenthesis indicate the fold change in band intensity relative to control. Note that the band intensities were normalized with their respective tubulin prior to the fold change calculation.

Transport of melanin-synthesizing enzymes such as TYRP1 (BLOC-1-dependent cargo) and TYR (AP-3-dependent cargo) is essential for melanosome biogenesis/maturation (Marks et al., 2013; Sitaram and Marks, 2012). We examined the pathway in which Rab9A participates and regulates cargo transport to melanosomes. IFM studies showed that TYRP1 colocalized with LAMP-2-positive structures in Rab9A-depleted melanocytes to a greater extent than in control shRNA-transduced cells (Figures4A,B and S3A). Furthermore, the lysosomal targeting of TYRP1 in Rab9A-knockdown cells was similar to that of the BLOC-3^−^ mutant cells, as well as Rab38, Rab32, or VARP-knockdown cells (Figures4A,B and  S3A; *r *=* *0.56 ± 0.03 in Rab9A sh, 0.67 ± 0.03 in BLOC-3^−^, 0.70 ± 0.02 in Rab38 sh, 0.63 ± 0.03 in Rab32 sh, and 0.62 ± 0.05 in VARP sh compared to 0.29 ± 0.02 in control sh) (Bultema et al., 2012; Gerondopoulos et al., 2012; Tamura et al., 2009; Wasmeier et al., 2006). Consistent with these results, TYRP1 protein levels were significantly reduced in Rab9A-depleted melanocytes compared to control cells, which is accordant with the TYRP1 levels in BLOC-3^−^ or Rab38, 32 or VARP-knockdown cells (Figure4C). Furthermore, TYRP1 levels were additionally reduced in Rab9A and Rab38 or Rab32 double knockdown cells (Figure S3C). In contrast, TYRP1 in BLOC-1^−^ or BLOC-2^−^ cells was partially targeted to lysosomes, while a subset of the protein mislocalized to endosomes, Golgi, and the cell surface (Figure S3B) (Dennis et al., 2015; Setty et al., 2007). Moreover, TYRP1 trafficking to melanosomes was unaffected in AP-3-deficient melanocytes (Figure S3B) (Setty et al., 2007). These results suggest that Rab9A regulates the transport of TYRP1 to melanosomes and functions in a similar manner as BLOC-3, Rab38, Rab32, and VARP proteins in melanocytes.

Next, we tested whether Rab9A is also required for the AP-3-dependent trafficking of TYR to melanosomes. IFM studies showed TYR in wild-type melanocytes localized to the limiting membranes of melanosomes. Surprisingly, in Rab9A-depleted melanocytes, TYR staining was reduced and appeared punctate, and few TYR-positive structures targeted to lysosomes (Figures4A and S3A). Similar to results in Rab9A-depleted cells, TYR staining was significantly reduced in BLOC-3^−^ cells, as well as in Rab38, 32, or VARP-knockdown cells (Figures4A and S3A). This was consistent with the reduced TYR protein levels in these cells (Figure4C). Furthermore, TYR levels were additionally reduced in Rab9A and Rab38 or Rab32 double knockdown cells (Figure S3C). The low TYR staining in these cells is likely due to faster degradation of the protein in the lysosomes. To confirm these results, we treated Rab9A, Rab38, or 32-depleted cells with bafilomycin A1 and it restored endogenous TYR staining to the lysosomal compartments (Figure S4A). Corroborating these results, transfection of GFP-TYR in Rab9A, Rab38, Rab32, VARP-knockdown, or BLOC-3^−^ cells showed more GFP-TYR in the lysosomes (LAMP-2-positive compartments) than in control cells (Figures S4B and 4B; *r *=* *0.59 ± 0.03 in Rab9A sh, 0.54 ± 0.05 in BLOC-3^−^, 0.62 ± 0.02 in Rab38 sh, 0.67 ± 0.03 in Rab32 sh, and 0.64 ± 0.02 in VARP sh compared to 0.24 ± 0.03 in control sh). These results suggest that TYR is mistargeted to lysosomes and degraded in the Rab9A, Rab38, Rab32-depleted, and BLOC-3^−^ melanocytes. Similarly, TYR staining was completely abolished in AP-3-deficinet melanocytes, consistent with previous reports suggesting that AP-3 is required for melanosomal transport of TYR (Figures S3A,B and 4A) (Honing et al., 1998; Theos et al., 2005). In contrast, TYR in BLOC-1^−^ and BLOC-2^−^ melanocytes accumulated in the Golgi and also localized as a punctate structures in the cell periphery (Figure S3B). Our previous studies have shown that a population of TYR correctly targets to melanosomes in BLOC-1^−^ and BLOC-2^−^ melanocytes (Figure S3B) (Dennis et al., 2015; Setty et al., 2007), indicating that BLOC-1, -2 and AP-3 alter TYR transport in a different way than Rab9A. Thus, these results indicate that Rab9A controls the transport of TYR to melanosomes in a manner similar to BLOC-3, Rab38, Rab32, or VARP proteins in melanocytes.

Because Rab9A, BLOC-3, Rab38, Rab32, and VARP are involved in both TYRP1 and TYR transport steps to melanosomes and their depletion leads to mistargeting of these proteins to lysosomes, we hypothesized that melanocytes lack the expression for these genes should accumulate stage II or immature melanosomes. Interestingly, the majority of PMEL, a stage II melanosome specific protein, colocalized with lysosomes in Rab9A, BLOC-3, Rab38, Rab32, or VARP-knockdown or mutant melanocytes, in contrast to its localization in control cells. This result suggests a block in the maturation of stage II melanosomes, which were then targeted to lysosomes (Figures4A,B and S3A; *r *=* *0.33 ± 0.03 in Rab9A sh, 0.31 ± 0.02 in BLOC-3^−^, 0.40 ± 0.04 in Rab38 sh, 0.40 ± 0.04 in Rab32 sh, and 0.44 ± 0.03 in VARP sh compared to 0.26 ± 0.04 in control sh). Similar to results in Rab9A-knockdown cells, lysosomal targeting of PMEL was observed in BLOC-1^−^ and AP-3^−^ melanocytes (Figure S3B). In contrast, a small population of PMEL in BLOC-2^−^ cells was colocalized or associated with LAMP-2-positive compartments, which was similar to its localization in control cells (Figure S3A,B). Furthermore, we tested whether the colocalization of PMEL with LAMP-2 is due the altered trafficking of LAMP-2 in BLOC-2^−^ cells. IFM studies in wild type or control shRNA-transduced cells showed LAMP-2 in ring-like structures dispersed in the peripheral cytosol, with a subpopulation colocalized with pigmented melanosomes (Figure S3D). This finding was consistent with a previous report (Raposo et al., 2001). As expected, the distribution of LAMP-2 compartments in the peripheral cytosol was not affected, but their colocalization with hypopigmented BF melanosomes increased modestly in Rab9A, BLOC-3, Rab38, Rab32, or VARP-knockdown or mutant melanocytes (Figure S3D). In contrast, LAMP-2 protein levels were slightly altered in these cells except BLOC-3^−^ melanocytes (Figure S3E), suggesting a possible role for Rab9A, Rab38, 32, and VARP in LAMP-2 trafficking in melanocytes. These results are consistent with increased surface levels of LAMP-1 and MPRs (mannose 6-phosphate receptors) observed in Rab9A-inactivated fibroblasts (Ganley et al., 2004). Moreover, BLOC-3-deficiency in melanocytes increased the perinuclear clustering of LAMP-2 compartments (arrow, Figure S3D), and this mirrors with previous observations in BLOC-3-deficient fibroblasts (Falcon-Perez et al., 2005). In addition, perinuclear distribution of LAMP-2 was significantly increased in BLOC-2^−^ cells (arrow, Figure S3D), similar to the localization of TYRP1 and TYR in these melanocytes (arrows, Figure S3B). Furthermore, our previous and unpublished studies showed an increase in the accumulation of LAMP-1 at the cell surface in BLOC-1^−^, -2^−^, or AP-3^−^ melanocytes (Setty et al., 2007), indicating that HPS deficiency slightly alters the trafficking of lysosomal proteins. Together, these results indicate that Rab9A regulates melanosome biogenesis by regulating cargo transport steps to melanosomes.

### Rab9A regulates the targeting of recycling endosome tubules to melanosomes

**Figure 5:**
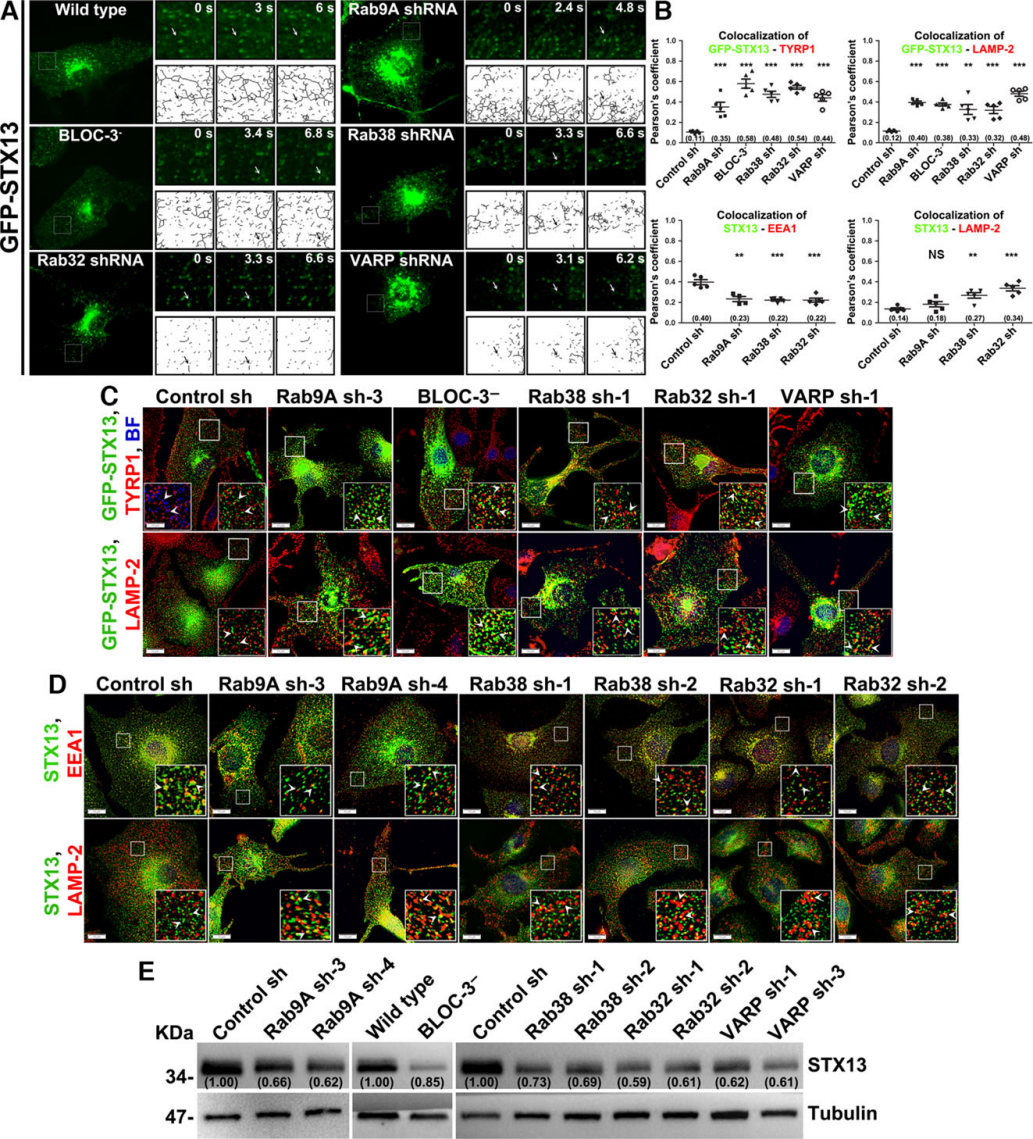
Live imaging of tubular recycling structures and their cellular distribution in Rab9A, Rab38/32, and VARP-knockdown or BLOC-3-deficient melanocytes. (A) Rab9A, Rab38/32 or VARP-knockdown or BLOC-3-deficiency in melanocytes alters the length of STX13-positive recycling endosomes. Gene knockdown or HPS-deficient melanocytes were transiently transfected with 2 μg of GFP-STX13 and then imaged by live microscopy. Insets represent GFP localization at different time points, and their respective skeleton images are shown separately. Arrows point to tubular GFP-STX13 structures. (B, C) Rab9A, Rab38/32 or VARP-knockdown or BLOC-3-deficient cells mislocalize the endosomal recycling SNARE, GFP-STX13, and melanosomal protein TYRP1 to lysosomes. Cells were transiently transfected with 2 μg of GFP-STX13, fixed, counter-stained with either TYRP1 or LAMP-2 and then analyzed by BF and IFM (C). Arrowheads point to GFP-STX13 localization. The colocalization efficiency between GFP-STX13 and TYRP1 or LAMP-2 was measured as Pearson’s correlation coefficient (*r*, n = ∼5–6 cells) and then plotted (B). (B, D) Rab9A or Rab38/32-depletion in melanocytes leads to partial mislocalization of endogenous STX13 to lysosomes. Knockdown melanocytes were fixed, stained for either the early endosomal marker EEA1 or the lysosomal marker LAMP-2, and then analyzed by IFM (D). Arrowheads point to STX13 localization. The colocalization efficiency between STX13 and EEA1 or LAMP-2 was measured as Pearson’s correlation coefficient (*r*, n = ∼5–6 cells) and then plotted (B). Values in parenthesis indicate the colocalization coefficient between the two marker proteins. Statistical significance in *r* value between control sh and Rab9A sh, Rab38/32 sh, VARP sh, or BLOC-3^−^ cells was measured using graphpad software. ns, not significant; &&, P < 0.01 and &&&, P < 0.001; mean±s.e.m. Nuclei were stained with Hoechst. Bars, 10 μm and insets, 2.5X of white boxed regions. (E) STX13 expression is significantly reduced in Rab9A, Rab38/32 or VARP-knockdown or BLOC-3-deficient melanocytes. Immunoblot analysis of cellular STX13 levels with *γ*-Tubulin as a loading control. Protein band intensities were quantified and normalized to *γ*-Tubulin. The fold change relative to control cells was then calculated as described in Supporting Information. Values in parenthesis indicate the fold change in band intensity relative to control. Note that the band intensities were normalized with their respective tubulin prior to the fold change calculation.

Rab GTPases localize to selective membranes and regulate membrane fusion events (Pfeffer, 2013; Stenmark, 2009). In addition, Rabs recruit specific effector proteins, and they sometimes interact with fusion machinery such as tethering factors and SNAREs used to bring membranes closer to each other (Kummel and Ungermann, 2014; Pfeffer, 2013). We tested whether Rab9A or Rab38/32 regulates endosomal recycling fusion events with melanosomes for cargo delivery. Electron tomographic and immunoelectron microscopic studies in melanocytes have shown that melanosomal proteins such as TYRP1 localize to the tubular recycling endosomal structures that are associated with the limiting membrane of melanosomes (Delevoye et al., 2009; Dennis et al., 2015), suggesting that melanosomes receive cargo through these tubular endosomal domains. In addition, these tubular structures are also positive for a recycling endosomal SNARE, STX13 (Dennis et al., 2015), which regulates the transport of both TYRP1 and TYR to melanosomes (Jani et al., 2015). We hypothesized that depletion of Rab9A or its co-regulators in melanocytes alters the dynamics of STX13-positive recycling endosomes. In wild-type melanocytes, GFP-STX13 localized to tubular endosomal structures that were persisted for a few seconds (Figure5A, skeletonized images shown separately, Video S2). In contrast, STX13-positive tubular structures were shorter in length in Rab9A-depleted cells, as well as in BLOC-3^−^, Rab38/32, or VARP-knockdown melanocytes compared to wild type or control shRNA-transduced cells (Figure5A, Videos S2–S7, and data not shown for control shRNA cells). This result is apparent in the skeletonized live images (arrows, Figure5A). Consistently, the number of tubular STX13 structures associated with melanosomes in wild-type cells was dramatically reduced in Rab9A-depleted or BLOC-3-deficient melanocytes (arrows, Figure S5A). These results suggest that Rab9A and its co-regulators are required either to maintain the length of STX13-positive recycling tubular structures, or for the stabilization of interaction between recycling tubular structures and melanosomes.

Next, we tested whether STX13-positive recycling endosomal structures are associated or colocalized with the melanosomal cargo TYRP1 in Rab9A knockdown cells. IFM analysis showed GFP-STX13 tubular structures in wild-type melanocytes were in close proximity to the pigment granules positive for TYRP1 (arrows, Figure5B, C, *r* = 0.11 ± 0.01). Surprisingly, the number of GFP-STX13 tubular structures was reduced in the Rab9A-depleted melanocytes, a result consistent with our findings from live imaging microscopy. In addition, STX13 appeared as enlarged punctate structures that were colocalized with TYRP1 (see below) in Rab9A-depleted melanocytes (Figure5B, C, *r* = 0.35 ± 0.05). Similarly, punctate GFP-STX13 was colocalized with TYRP1 in BLOC-3^−^, Rab38, Rab32, or VARP knockdown or mutant cells (Figure5B,C; *r *=* *0.58 ± 0.05 in BLOC-3^−^, 0.48 ± 0.03 in Rab38 sh, 0.54 ± 0.02 in Rab32 sh and 0.44 ± 0.03 in VARP sh). As shown in Figures4A and S3A, TYRP1 in these knockdown or mutant cells localized to lysosomes. To test whether the lysosomal targeting of TYRP1 or TYR is mediated through STX13, we investigated the localization of the SNARE in Rab9A-knockdown cells. IFM analysis showed that GFP-STX13 was targeted to lysosomes in Rab9A-depleted cells, as well as in BLOC-3^−^, Rab38, Rab32, or VARP-knockdown or mutant cells. This was consistent with the localization of GFP-STX13 to LAMP-2-positive structures (Figure5B,C; *r *=* *0.4 ± 0.01 in Rab9A sh, 0.38 ± 0.02 in BLOC-3^−^, 0.33 ± 0.05 in Rab38 sh, 0.32 ± 0.04 in Rab32 sh and 0.48 ± 0.03 in VARP sh). As expected, GFP-STX13 in control cells localized to recycling endosomal structures and partly associated with melanosomal TYRP1 (Setty et al., 2008), but did not target to lysosomes (Figure5B,C; *r *=* *0.12 ± 0.01). Furthermore, we examined these results by analyzing the steady-state localization of STX13 with respect to lysosomal or endosomal markers (LAMP-2 or EEA1, respectively). Endogenous STX13 partially localizes to EEA1-positive early endosomes (*r *=* *0.4 ± 0.03) (Setty et al., 2008), but not to LAMP-2-positive structures in wild-type melanocytes (Figure5B,D; *r *=* *0.14 ± 0.01). STX13 localization to lysosomes significantly increased in Rab9A, 38, and 32-knockdown cells (using two different shRNAs; *r *=* *0.18 ± 0.03 in Rab9A sh, 0.27 ± 0.03 in Rab38 sh and 0.34 ± 0.03 in Rab32 sh), and its colocalization with EEA1 was correspondingly reduced in these cells (Figure5B,D; *r *=* *0.23 ± 0.03 in Rab9A sh, 0.22 ± 0.01 in Rab38 sh and 0.22 ± 0.02 in Rab32 sh). Moreover, these results were consistent with the reduced STX13 protein levels in Rab9A, Rab38/32, VARP-knockdown or BLOC-3^−^ melanocytes relative to control cells (Figure5E), suggesting that Rab9A and its co-regulators play a crucial role in targeting STX13-positive recycling tubular structures to melanosomes. GFP-STX13 localized to vacuolar endosomal structures and colocalized with mislocalized endosomal TYRP1 in BLOC-1^−^ and BLOC-2^−^ melanocytes, whereas STX13 colocalized with melanosomal localized TYRP1 in AP-3-cells (Jani et al., 2015; Setty et al., 2007), in contrast to wild-type melanocytes (Figure S5B). Accordingly, STX13 levels were unaffected in BLOC-1^−^ and BLOC-2^−^ cells, and only slightly reduced in AP-3^−^ melanocytes (Figure S5C), indicating that BLOC-1, BLOC-2, or AP-3 complexes regulate cargo transport to melanosomes upstream of STX13 function. Together, these results indicate that STX13 together with melanosome cargo are targeted to lysosomes in the absence of Rab9A or its co-regulators.

STX13 contains a regulatory (Habc) domain (14–129 aa), similar to the other syntaxin family members (Hong, 2005). Our recent studies have shown that a mutant STX13 missing the regulatory domain (myc-STX13^Δ129^, active SNARE) actively participates in melanosome transport steps and increases the delivery of TYRP1 and TYR to melanosomes. This in turn increases melanocyte pigmentation (Jani et al., 2015). In addition, the myc-STX13^Δ129^ mutant has been shown to mislocalize to melanosomes in wild-type melanocytes (Jani et al., 2015). We therefore tested whether overexpression of active SNARE, STX13 could rescue the hypopigmentation of, or defective cargo delivery to melanosomes in Rab38 or 32-depleted melanocytes. As expected, myc-STX13^Δ129^ localized to the melanosomes (arrows), but not to lysosomes (arrowheads) in melanocytes transduced with control shRNA (Figure S5D). In addition, expression of STX13^Δ129^ did not rescue the pigmentation deficit of Rab38 or 32-knockdown cells (Figure S5D). Moreover, localization of myc-STX13^Δ129^ to hypopigmented melanosomes was drastically reduced (arrows), and it colocalization with lysosomes significantly increased (arrowheads), upon Rab38 or Rab32-knockdown (Figure S5D). These results suggest that Rab38 or 32 are required for the function of active STX13 SNARE. Together, these results suggest that Rab9A, 38, 32, BLOC-3, and VARP regulate the targeting of STX13-positive recycling tubular endosomes to melanosomes.

## Discussion

**Figure 6:**
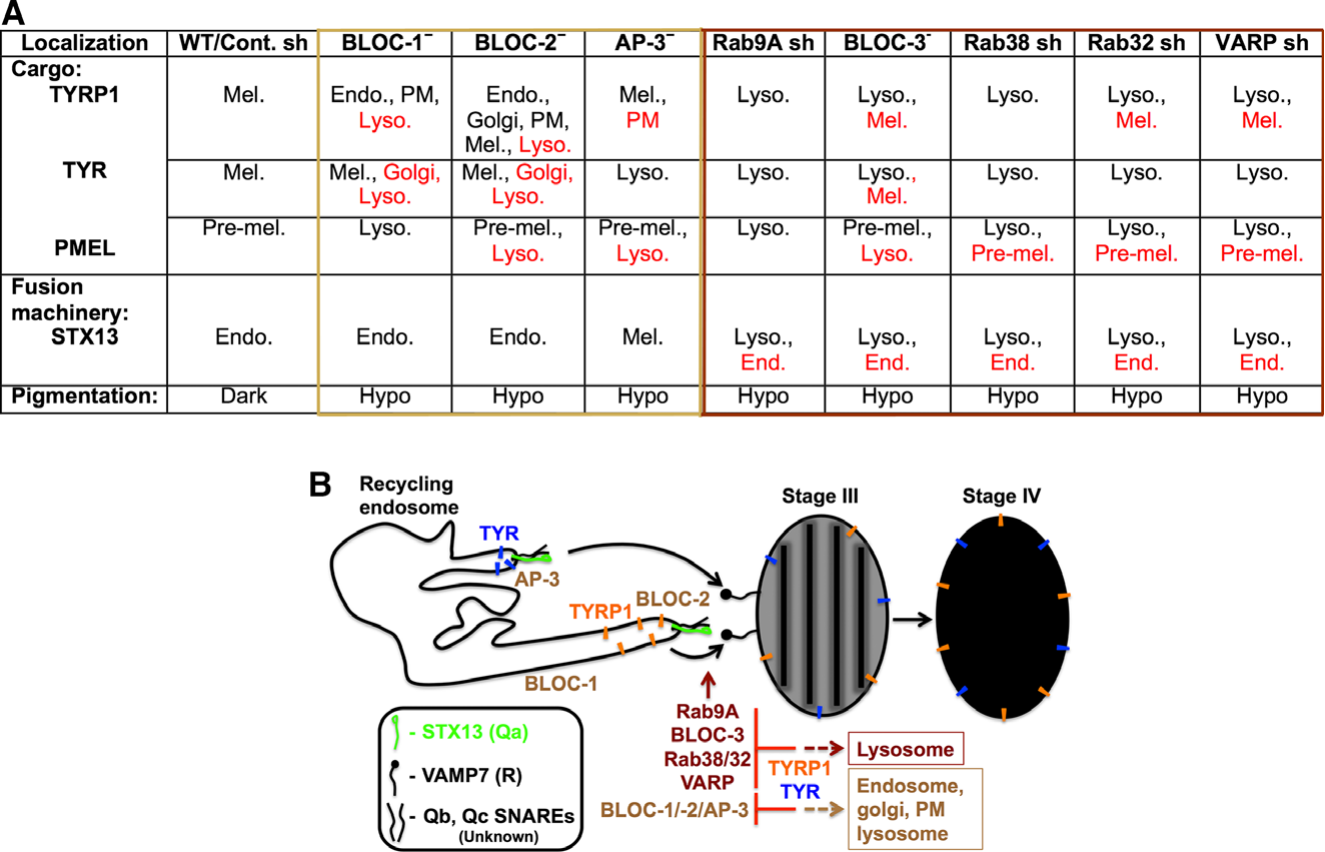
Model depicting the function of Rab9A and its co-regulators and HPS complexes in melanosome cargo transport and pigmentation. (A) Cellular pigmentation and localization of melanosomal cargo and endosomal SNARE in the shRNA-depleted or HPS mutant melanocytes. Cells were fixed, stained, and analyzed by IFM. Melanocyte pigmentation was analyzed by BFM. Organelle-specific markers were used to study the distribution of cargo or STX13 in all cells. Red text indicates a subset of cargo or SNARE localization to the indicated organelles. Mel., Melanosomes; Pre-mel., stage II melanosomes; Endo., endosomes; Lyso., lysosomes; PM, plasma membrane; Dark, normal pigmentation; and Hypo, hypopigmentation. Note that the localization of melanosomal cargo in BLOC-1^−^, BLOC-2^−^, and AP-3^−^ cells was characterized extensively in (Dennis et al., 2015; Setty et al., 2007; Theos et al., 2005). (B) Schematic representation of cargo transport from recycling endosomes to melanosome through STX13-mediated membrane fusion. Model representing the cargo TYRP1 (BLOC-1-dependent cargo, orange symbols) and TYR (AP-3-dependent cargo, blue symbols) transport from tubular-vesicular structures of recycling endosomes during melanosome biogenesis. STX13 (Qa SNARE, green) with two other unknown SNAREs (Qb, Qc; black) on recycling endosomes makes *trans*-SNARE complex with VAMP7 (R-SNARE, black) on Stage III or Stage II (not shown) melanosome during cargo delivery. Upon cargo transport and melanin synthesis, Stage III matures into Stage IV melanosome. Curved arrows represent the fusion of endosomal transport carriers with melanosomes. Dotted arrows point to the mislocalization of cargo to other organelles in the absence or deficiency (represented as inhibition symbol, red) of respective molecules or complexes. The solid black lines in the stage III melanosome represent the PMEL fibrils. Based on the data presented here and previous literature, BLOC-1 and AP-3 possibly function at early and BLOC-2/Rab9A/BLOC-3/Rab38/32/VARP later stages of cargo transport to the melanosome.

Mammalian cells produce a variety of organelles, including late endosomes and lysosomes which are formed through sequential maturation of endosomes via cargo transport and membrane fusion events (Kummel and Ungermann, 2014). Specialized cells such as melanocytes utilize recycling endosomes for the delivery of melanin-synthesizing enzymes to melanosomes. This process is dependent on the HPS complexes BLOC-1, -2, -3, and AP-3 (Marks et al., 2013; Sitaram and Marks, 2012). Although several Rab GTPases are known to regulate melanosome cargo transport, our study identified a new function for Rab9A in LRO biogenesis, separate from its known role in retrograde transport of cargo from late endosomes to Golgi in fibroblasts. Moreover, our study suggested that Rab9A works in a similar manner as BLOC-3, Rab38/32, and VARP in the melanosome biogenesis pathways (summarized in Figure6A).

The melanosomal cargoes TYRP1 and TYR follow two different routes from tubular recycling endosomal structures to melanosomes (Marks et al., 2013; Sitaram and Marks, 2012). Our results showed that Rab9A and its co-regulators, BLOC-3, Rab38/32, and VARP, are essential for both cargo transport steps, and therefore also for melanosome biogenesis and pigmentation (Figure6A). As previously reported and confirmed here, mutations in the subunits of HPS complexes such as BLOC-1 or BLOC-2 lead to mislocalization of the TYRP1 either to multiple organelles of the biosynthetic pathway or to lysosomes for degradation. In addition, a subset of TYRP1 correctly targets to melanosomes in BLOC-2^−^ cells (Figure S3) (Dennis et al., 2015; Di Pietro et al., 2006; Setty et al., 2007). Conversely, AP-3 deficiency in melanocytes misroutes the transport of TYR but not TYRP1 to melanosomes (Figure S3) (Chapuy et al., 2008; Huizing et al., 2001; Theos et al., 2005), and thus inhibits pigment granule maturation in HPS-deficient melanocytes. However, our study indicates that Rab9A, BLOC-3, Rab38/32, or VARP control the transport of both TYRP1 and TYR by regulating the targeting of STX13-positive recycling tubules to melanosomes (summarized in Figure6A). In addition, the inactivation or deficiency of these molecules in melanocytes targets premelanosomes to lysosomes that block pigment granule maturation (Figures5 and Figure S3). Targeting of PMEL to lysosomes presumably occurs through increased autophagy in BLOC-3^−^ cells (Smith et al., 2005), or in Rab9A (Nozawa et al., 2012) or Rab32 (Hirota and Tanaka, 2009)-knockdown cells because these molecules have a role in autophagosome biogenesis. Alternatively, non-functional premature melanosomes are degraded through basal autophagy in melanocytes (Ho and Ganesan, 2011). These results indicate a model (Figure6B) wherein Rab9A, BLOC-3, Rab38/32, and VARP function in a linear pathway and work downstream of BLOC-1 and AP-3 complexes. Several observations support our model: (i) knockdown of Rab9A, Rab38/32, or VARP, or mutations in BLOC-3 subunits resulted in hypopigmented melanosomes due to mistargeting of melanosomal cargo to lysosomes; (ii) deficiency of BLOC-1, -2, or AP-3 led to the mislocalization of cargo to multiple cellular structures other than lysosomes; (iii) inactivation of Rab9A and its co-regulators affected the length of STX13-positive tubular structures and mislocalized the SNARE to lysosomes; and (iv) BLOC-1 or BLOC-2-deficiency affected the structure of STX13-positive recycling endosomes (Dennis et al., 2015; Setty et al., 2007) and retained the SNARE in endosomes (Setty et al., 2008). Thus, these pieces of evidence support a role for Rab9A and its co-regulators BLOC-3, Rab38/32, and VARP in targeting recycling endosomes to maturing melanosomes.

Our model (Figure6B) is also consistent with the hypothesis that BLOC-1, -2, and AP-3 complexes function upstream of Rab9A or its co-regulators, presumably regulating the initial transport or sorting steps on recycling endosomal domains (Delevoye et al., 2009; Setty et al., 2007, 2008; Theos et al., 2005). This is in accordance with the absence of tubular endosomal domains and the accumulation of cargo in vacuolar endosomes in BLOC-1-deficient melanocytes (Delevoye et al., 2009; Setty et al., 2007). Moreover, recycling endosome tubules are shorter in length in BLOC-2-deficient cells, similar to the lengths observed in Rab9A or Rab38/32 depleted melanocytes, and cargo accumulated in the Golgi and at the cell surface in these cells (Dennis et al., 2015). However, we predict that these tubular structures are not affected in AP-3-deficient cells (Figure S5), as TYRP1 localizes to the melanosomes in these cells (Figure S3B) (Theos et al., 2005). In contrast, our results showed that Rab9A, BLOC-3, or Rab38/32 depletion mistargeted these tubular structures to lysosomes, indicating that these molecules function downstream of BLOC-1 or AP-3 complexes (Figure6A). Alternatively, these molecules might also closely work with BLOC-1, -2, or AP-3 because an interaction has been previously noted between Rab38/32 and HPS complexes (Bultema et al., 2012).

How Rab9A and its co-regulators work together with HPS complexes for accurate targeting of cargo to melanosomes remains an open question. We hypothesize that the BLOC-1-AP-1-KIF13A complex, along with actin cytoskeleton regulators such as the WASH complex (Delevoye et al., 2009; Ryder et al., 2013), generates the tubular recycling structures into which TYRP1 and other melanosomal cargoes are sorted by AP-1 (Chapuy et al., 2008). Furthermore, these tubular structures are stabilized or tethered by the BLOC-2 complex (Dennis et al., 2015). In contrast, sorting of TYR into vesicular or tubular domains is not well understood, and we predict that AP-3 and an unknown motor protein coordinate to generate these structures. However, to our surprise, BLOC-2^−^ melanocytes clustered or accumulated TYR at the perinuclear region, similar to the localization of TYRP1 (Figure S3) and suggesting that BLOC-2 functions in both BLOC-1- and AP-3-dependent transport steps. Interestingly, BLOC-2 interacts more strongly with Rab38 than Rab32 (Bultema et al., 2012), suggesting that BLOC-2 and Rab38 function in directing the recycling endosomes to melanosomes. Surprisingly, depletion of Rab38 or its genetic compensator Rab32 misroutes TYRP1 and TYR to lysosomes in melanocytes (Figures4, S3 and S4). This is different than results in BLOC-2-deficient cells (Figure S3B), indicating that Rab38/32 functions downstream of the BLOC-2 complex. Furthermore, we presume that Rab9A helps in recruiting Rab38/32 onto the endosomal or melanosomal membranes through a GEF, BLOC-3, a hypothesis that is consistent with 1) the known interaction of Rab9A with the HPS4 subunit of BLOC-3 (Kloer et al., 2010) and 2) the targeting of cargo to lysosomes when Rab9A is inactivated (Figures4 and S3). However, Rab38 localizes to STX13-positive endosomes in the Rab9A-knockdown cells (Figure3) indicating that recruitment of BLOC-3 to these membranes may be independent of Rab9A. Nevertheless, the precise mechanism of Rab9A-dependent membrane recruitment of BLOC-3 in melanocytes needs to be addressed in future. Overall, Rab9A mediates the recruitment of Rab38/32 through BLOC-3, which then interacts with BLOC-2. Alternatively, Rab9A-BLOC-3-Rab38/32 may be recruited to membranes in sequential steps during endosomal fusion with melanosomes, where Rab38 interacts with BLOC-2 and/or SNARE proteins. The latter possibility is consistent with the observation that STX13-positive tubules in Rab9A, BLOC-3, Rab38/32 knockdown, or mutant cells are shorter than in control cells (Figure5), similar to the phenotype of BLOC-2^−^ cells (Dennis et al., 2015). In addition, our recent studies have shown that SNAREs such as STX13 and VAMP7 are interdependent in regulating the cargo trafficking to melanosomes, and their inactivation presumably blocks the fusion of tubular recycling structures with melanosomes (Jani et al., 2015). It is unknown whether Rab9A or Rab38/32 interacts directly or indirectly with STX13, but deficiency in either Rab leads to mislocalization of the SNARE to lysosomes (Figure5). This suggests that Rab9A and its co-regulators control STX13-mediated cargo transport. Interestingly, Rab38 also interacts with VARP (Wang et al., 2008), a protein that localizes to melanosomes (Tamura et al., 2009) and other organelles (Zhang et al., 2006), and that regulates the fusion activity of VAMP7 (Burgo et al., 2009; Schafer et al., 2012). Based on these studies, we predict that the Rab38-VARP interaction may facilitate the interaction of STX13 and VAMP7 during tubular endosome fusion with melanosomes. This hypothesis is consistent with the localization of Rab38 (Figure3) (Bultema et al., 2012), VARP (Tamura et al., 2009) and VAMP7 (Bultema et al., 2014; Jani et al., 2015) to melanosomes, as well as with the finding that the intermolecular interaction between Rab38 and VARP regulates TYRP1 transport to melanosomes (Tamura et al., 2011). Thus, Rab38 may function on both recycling endosomal membranes and melanosome membranes to interact with BLOC-2 and VARP, respectively, indicating its key role in melanosome biogenesis.

Surprisingly, Rab9A overexpression could neither rescue the pigmentation phenotype caused by Rab38-inactivation nor could Rab38 overexpression compensate for loss of Rab9A. This suggests an interdependent regulation between these Rabs for melanosome biogenesis (Figure S2). However, these Rabs appear to localize independently to recycling domains or melanosome membranes (Figures3). Owing to the difficulty in localizing the cytosolic BLOC-3 complex in Rab9A-knockdown cells and to the unknown mechanism of Rab9A recruitment (Yoshimura et al., 2010), we presume that Rab9A and Rab38 communicate with each other either through BLOC-3 or an unknown factor on the same membrane. Moreover, the precise role of Rab32 is not clear at this time. In addition, our studies showed that Rab9A primarily localized to lysosomes (Figure1), and partially to endosomes and melanosomes in melanocytes, similar to its localization to late endosomes in fibroblasts (Lombardi et al., 1993). Interestingly, inactivation of Rab9A, 38/32, or VARP leads to reduced LAMP-2 levels, similar to what is observed in BLOC-3^−^ cells (Figure S3) and suggesting that Rab9A regulates the trafficking of both lysosomal and melanosomal cargo in melanocytes (Chia et al., 2011; Lombardi et al., 1993). This explanation is consistent with the idea that Rabs can have multiple roles based on their membrane or organelle localization (Stenmark, 2009). Overall, our work demonstrated that Rab9A and its co-regulators influence melanosome biogenesis by regulating the STX13-mediated cargo transport steps to melanosomes.

## Methods

### Cell lines and cell culture

Immortal mouse melanocytes used in this study were derived from wild-type (melan-Ink4a) or HPS-deficient mouse models (BLOC-1^−^, melan-mu; BLOC-2^−^, melan-coa; BLOC-3^−^, melan-le and AP-3^−^, melan-mh). Detailed information on cell lines, culture conditions, transfection, and transduction is described in the Supporting Information.

### Immunofluorescence microscopy and image analysis

Cells were fixed and stained as described previously (Setty et al., 2007). BFM and IFM were performed using an Olympus IX81 motorized inverted fluorescence microscope equipped with 60X oil immersion U Plan super apochromat objective and a CoolSNAP HQ2 CCD camera (Photometrics, Tucson, AZ, USA). Images were deconvolved and analyzed with the cellSens Dimension package with the 5D module. Melanocytes were visually quantified as normal or hypopigmented by counting ∼100 cells in each experiment (n = 3) from BF images that were taken randomly from the sample using identical camera settings. Average cell pigmentation was calculated and then plotted. The colocalization coefficient between two colours was measured by selecting equal area randomly covering an entire cell, except for its perinuclear area, and then estimating the Pearson’s correlation coefficient (*r*) value using cellsens dimension software. Average ‘*r*’ values were calculated from 5 to 6 cells and then plotted. The analyzed images were assembled using Adobe Photoshop.

### Live cell imaging of GFP-STX13 and GFP-Rab9A in melanocytes

Melanocytes were plated on 2-cm glass-bottom dishes (MatTek Corp., Ashland, OR, USA) and then transfected with the GFP fusion construct. After 48 h of transfection, cells were visualized with an Olympus IX81 fluorescence microscope equipped with an environmental chamber maintained at 37°C with 5% CO_2_. Video microscopy of GFP or time-lapse imaging of GFP and BF melanosomes was performed by capturing image streams over 3–5 min using a CoolSNAP HQ2 CCD camera (Photometrics). Images were analyzed with the cellsens dimension software and processed into binary and then skeletonized using imagej software (NIH).

### Statistical analysis

Statistical significance was determined by the unpaired Student’s t-test and variance analysis using graphpad software. All values were described as mean±s.e.m. ns, not significant; &, P < 0.05; &&, P < 0.01 and &&&, P < 0.001.

## Supporting Information

**Figure S1.** Subcellular fraction and the effect of Rab9A expression in wild-type melanocytes.

**Figure S2.** ShRNA mediated knockdown and pigmentation analysis, and BFM of melanocytes.

**Figure S3.** Localization and steady-state levels of melanosomal or lysosomal proteins in Rab9A, Rab38/32 or VARP-knockdown (2^nd^ shRNA), or BLOC-1/-2/-3 or AP-3-deficient melanocytes.

**Figure S4.** Localization of endogenous TYR after bafilomycin treatment or GFP-TYR to lysosomes in Rab9A or Rab38/32-depleted melanocytes.

**Figure S5.** Time-lapse imaging of recycling tubular structures in Rab9A-knockdown or BLOC-3-deficient melanocytes, and localization of GFP-STX13^WT^ or GFP-STX13^Δ129^ in melanocytes deficient for BLOC-1/-2 or AP-3 or knockdown for Rab38/32.

**Video S1.** Time-lapse imaging of GFP-Rab9A in wild-type (melan-Ink4a) melanocytes.

**Video S2.** Video microscopy of GFP-STX13 in wild-type (melan-Ink4a) melanocytes.

**Video S3.** Video microscopy of GFP-STX13 in Rab9A sh-3 transduced wild-type melanocytes.

**Video S4.** Video microscopy of GFP-STX13 in BLOC-3-deficient melanocytes.

**Video S5.** Video microscopy of GFP-STX13 in Rab38 sh-1 transduced wild-type melanocytes.

**Video S6.** Video microscopy of GFP-STX13 in Rab32 sh-1 transduced wild-type melanocytes.

**Video S7.** Video microscopy of GFP-STX13 in VARP sh-1 transduced wild-type melanocytes.
