# Supplementary figures and images for: Rab9A is required for delivery of cargo from recycling endosomes to melanosomes
Source: Pigment Cell Melanoma Res. 2015 Dec 15;29(1):43–59. doi: 10.1111/pcmr.12434 (PMC4690521; doi:10.1111/pcmr.12434)

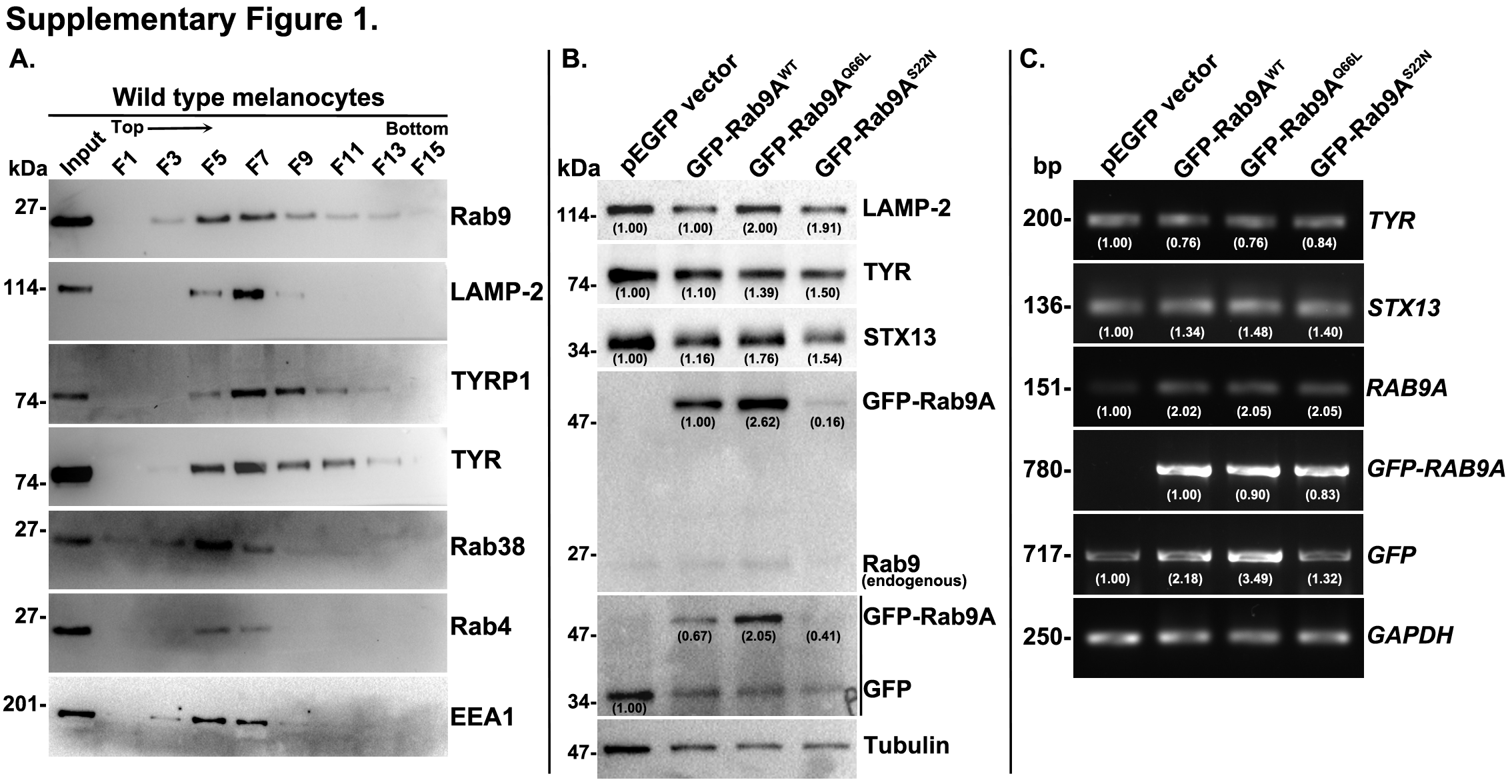

Supplement: Supplementary file 1 [file pcmr0029-0043-sd1.tif]

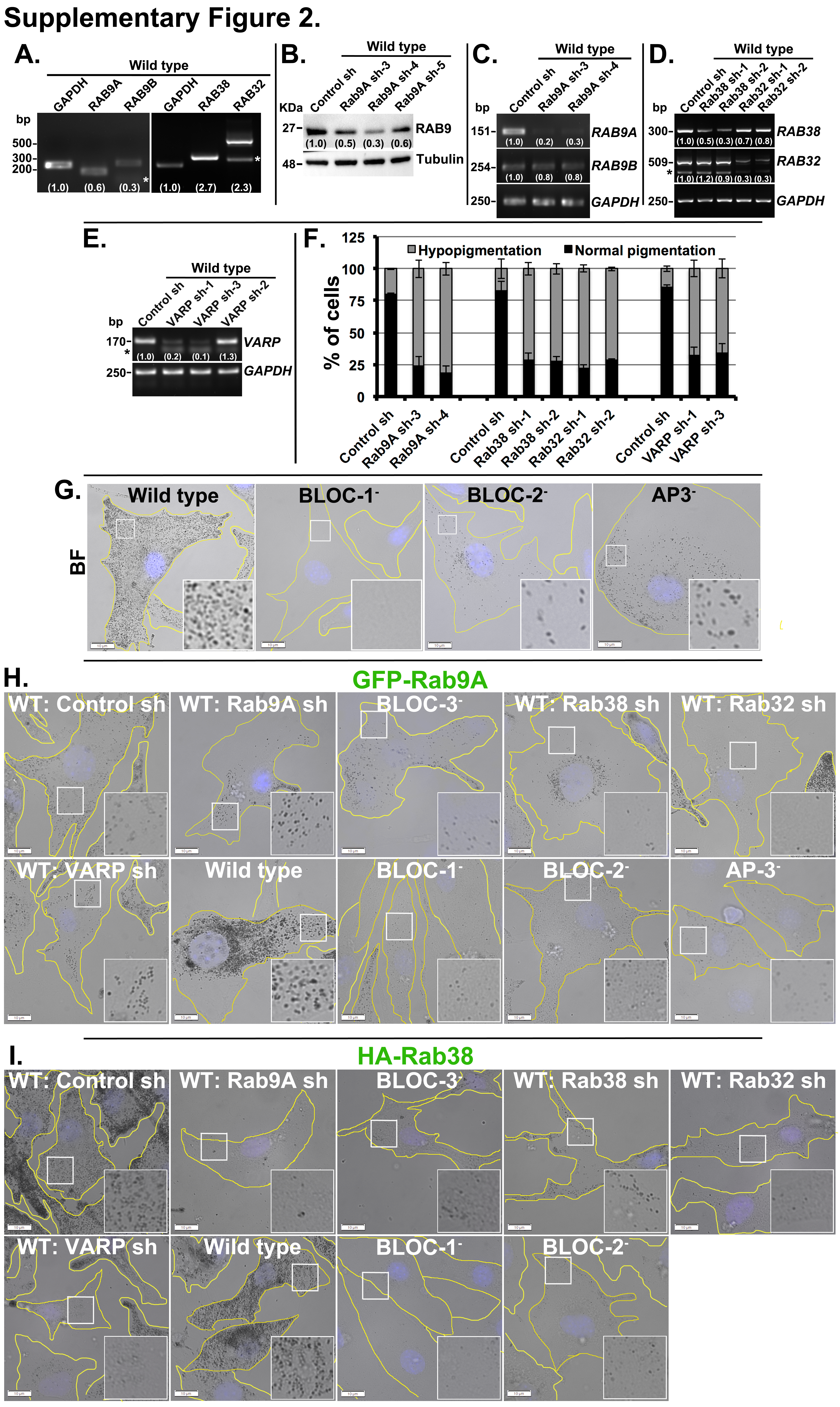

Supplement: Supplementary file 2 [file pcmr0029-0043-sd2.tif]

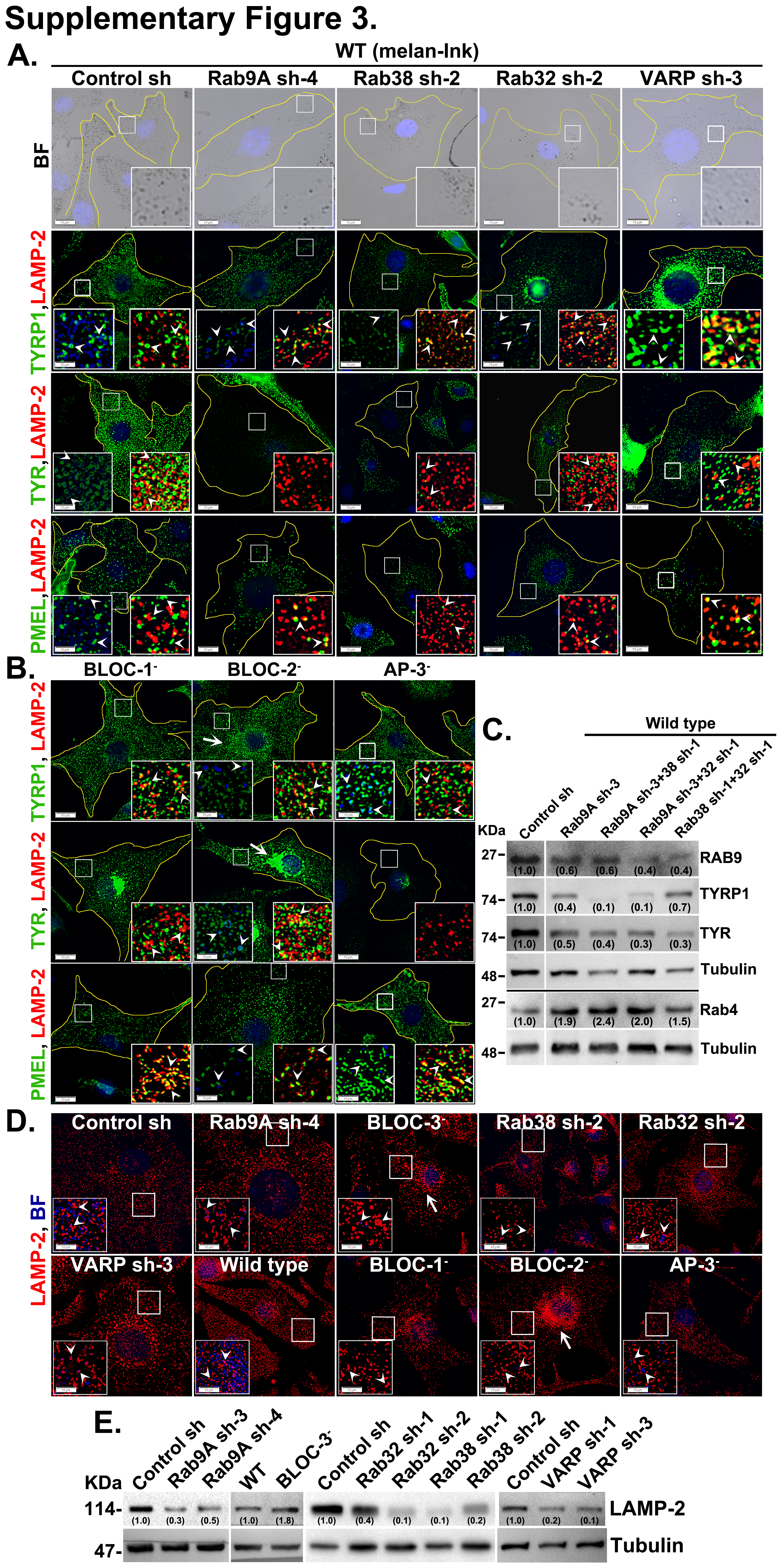

Supplement: Supplementary file 3 [file pcmr0029-0043-sd3.tif]

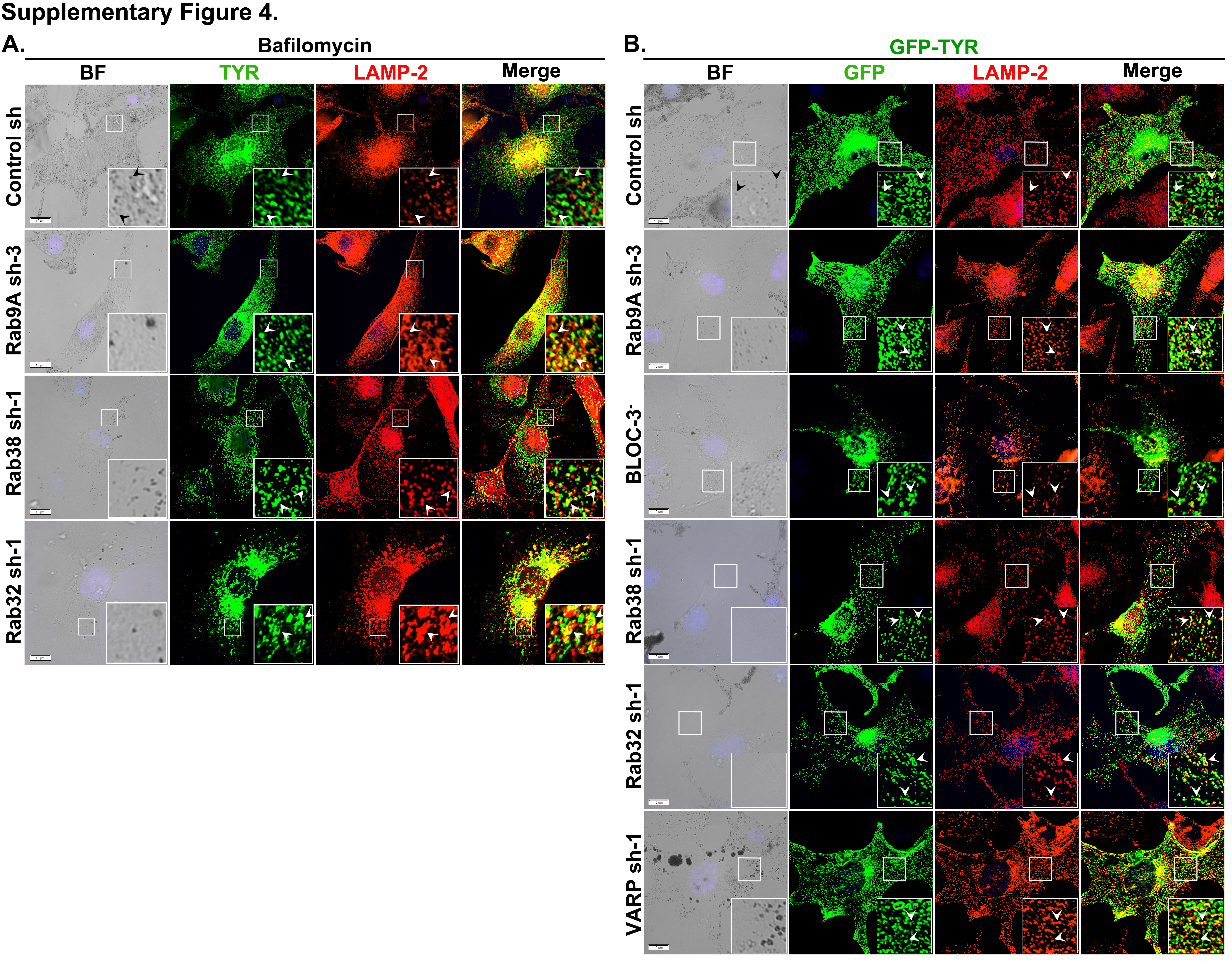

Supplement: Supplementary file 4 [file pcmr0029-0043-sd4.tif]

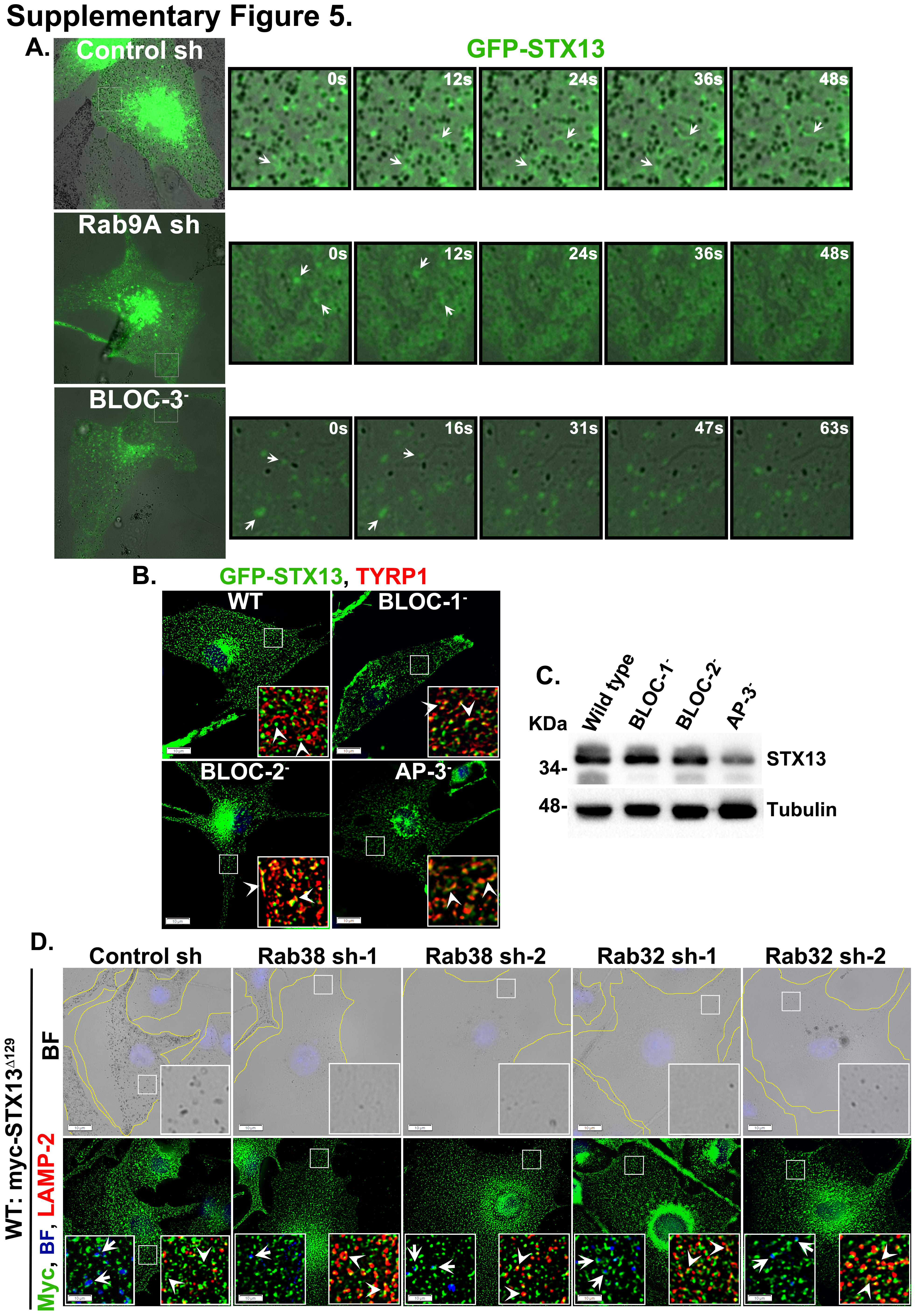

Supplement: Supplementary file 5 [file pcmr0029-0043-sd5.tif]
