# Supplementary material for: Rab9A is required for delivery of cargo from recycling endosomes to melanosomes
Source: Pigment Cell Melanoma Res. 2015 Dec 15;29(1):43–59. doi: 10.1111/pcmr.12434 (PMC4690521; doi:10.1111/pcmr.12434)
Supplement: Supplementary file 13 [file pcmr0029-0043-sd13.docx]

**Supplementary Information**

**Title: Rab9A is required for delivery of cargo from recycling endosomes to melanosomes**

Mahanty and Ravichandran *et al.*,

**Supplementary Materials**

Chemicals and tissue culture reagents were purchased from Sigma-Aldrich or Life Technologies (Invitrogen).

**Antibodies**

Polyclonal antisera to Rab5 and Rab9 (Cell Signaling Technology), Rab38 (Protein Tech), GFP (Invitrogen), TYRP1 (H-90, for immunoblotting) and EEA1 (goat, Santa Cruz Biotechnology) were used. Other antisera to STX13 ([Prekeris et al., 1998](#_ENREF_6)) and TYR ([Theos et al., 2005](#_ENREF_12)) have been previously described. Monoclonal antibodies to Rab4 (BD Biosciences), PMEL (Abcam, HMB45), TYRP1 (ATCC, TA99, for IFM), LAMP-2 (GL2A7) and c-Myc (9E10, Developmental Studies Hybridoma Bank), γ-Tubulin and HA (Sigma-Aldrich) were used. Secondary antibodies were either from Molecular Probes (Life Technologies) or Jackson Immunoresearch.

**DNA and shRNA constructs**

Expression constructs: GFP-Rab9A (also referred as GFP-Rab9A^WT^) ([Choudhury et al., 2002](#_ENREF_1)) and GFP-Tyrosinase (TYR) ([Halaban et al., 2000](#_ENREF_4)) were obtained from Addgene. GFP-Rab9A^Q66L^ and GFP-Rab9A^S22N^: Mutagenesis of amino acids glutamine (Q) at 66 position to leucine (L) and serine (S) at 22 position to asparagine (N) in GFP-Rab9A^WT^ was carried out using QuickChange multi site-directed mutagenesis kit (Agilent Technology). HA-Rab38: Human *RAB38* was PCR amplified with an N-terminal HA-epitope tag primer, digested with SalI and XbaI enzymes, and subcloned into the XhoI and XbaI sites of pcDNA3.1 (+) (Invitrogen). GFP-STX13 or GFP-STX13^WT^: Full length human *STX13* was PCR amplified ([Setty et al., 2008](#_ENREF_7)), digested with BamH1 and XhoI enzymes and subcloned into the BglII and SalI sites of pEGFP-C1 (Clontech). Myc-STX13^Δ129^: Human *STX13* lacking the sequence encoding the first 129 N-terminal amino acids was PCR amplified with a N-terminal myc-epitope primer and subcloned into the BamH1 and XhoI sites of pcDNA3.1(+) (Jani et al., 2015).

ShRNA vectors: Oligodeoxyribonucleotide duplexes containing the target gene sequence were cloned into the BamH1 and HindIII sites of the pRS shRNA vector (OriGene Technologies). The following sequences were selected as targets: CACCGAAAGCCCAAGCCAA (Rab9A sh-3), Gcagaagagttcatttactaa (Rab9A sh-4), TCTGGAGGTGGACGGACATTT (Rab9A sh-5), CCTCTGCCAAGGATAATATAA (Rab32 sh-1), ACATGCTTGCAAACCAGCAAAGTTT (Rab32 sh-2), GAGTCTATAGAACCGGACATT (Rab38 sh-1), CAAGAGTTTATTACCGGGAAGCTAT (Rab38 sh-2), GATTTGGCGACAGACTATTTC (VARP sh-1), ACCACATAGACTCCGTAAATG (VARP sh-2) and AAGATGTGCCACCCACTTTGT (VARP sh-3). Empty pRS shRNA plasmid was used as a control (referred to here as “Control sh”) in all shRNA knockdown experiments. Based on the efficiency of knockdown in wild type melanocytes (Supplementary Figure 2), Rab9A sh-3, Rab9A sh-4, VARP sh-1 and VARP sh-3 were used for IFM analysis.

**Supplementary Methods**

**Cell culture, transfection and retroviral transduction**

Immortal melanocyte cell lines: wild type melan-Ink4a-Arf-1 (from C57BL/6J *Ink4a-Arf^-/-^* mice*,* formerly called melan-Ink4a-1, referred to here as WT or melan-Ink4a) ([Ha et al., 2007](#_ENREF_3)), BLOC-1-deficient melan-mu1 (from B6/CHMU/Le *Mu^mu/mu^* mice, referred to here as BLOC-1^-^ or melan-mu) ([Setty et al., 2007](#_ENREF_8)), BLOC-2-deficient melan-coa2 (from C57BL/10J *Hps3^coa^*/*^coa^* mice, referred to here as BLOC-2^-^ or melan-coa) ([Suzuki et al., 2001](#_ENREF_10)), BLOC-3-deficient melan-le1 (from C57BL/6J *Hps4^le^*/*^le^* mice, referred to here as BLOC-3^-^ or HPS4-deficient) ([Suzuki et al., 2002](#_ENREF_9)) and AP-3-deficient melan-mh1 (from C57BL/6J *Ap3d^mh^*^/^*^mh^* mice, referred to here as AP-3^-^ or melan-mh) were maintained as previously described ([Sviderskaya et al., 2002](#_ENREF_11)).

Melanocytes or PLAT-E cells (Cell Biolabs) were transfected with plasmids using Lipofectamine 2000 (Invitrogen) according to the manufacturer’s protocol. ShRNA-encoding retroviruses were isolated from PLAT-E cells ([Morita et al., 2000](#_ENREF_5)). Melanocytes were transduced with retrovirus ([Setty et al., 2007](#_ENREF_8)) and selected twice with puromycin (1 or 2 μg/ml) on the 3rd and 5th day of transduction. For IFM and live cell imaging experiments, shRNA knockdown cells or wild type melanocytes were transiently transfected with expression plasmids using Lipofectamine 2000 and visualized after 48 h.

**Protease inhibitor assay**

Cells grown on a Matrigel-coated coverslip were treated with 50 nM bafilomycin A1 for 4 h at 37 ^0^C. Post treatment, cells were washed, fixed, stained and analyzed by IFM.

**Estimation of melanin pigments**

The melanin pigments from mouse melanocytes were estimated using a protocol described previously ([Wasmeier et al., 2006](#_ENREF_47)). Cells were lysed by sonication in lysis buffer (50 mM Tris-HCl pH 7.4, 2 mM EDTA, 150 mM NaCl, 1X protease inhibitors) and then pelleted, washed once with 1:1 ethanol and diethyl ether mixture. Further, pellet was air dried, solubilized in a buffer (2M NaOH, 20% DMSO) at 60 ^0^C for 30 min. and then measured the absorbance at 492 nm. The results were normalized to protein concentration and then plotted as fold change relative to the control.

**Subcellular fractionation**

Wild type (melan-Ink) melanocytes were washed with PBS, suspended in 0.25 M sucrose buffer (0.25 M sucrose, 1 mM EDTA, 25 mM HEPES, pH 7.4, 0.02% sodium azide and protease inhibitor cocktail) and then homogenized using Dounce homogenizer. Cell lysate was centrifuged at 600*g* at 4 ^0^C for 10 min and fractionated on a sucrose step gradient (2.0 M, 1.6 M, 1.4 M and 1.2 M sucrose densities from bottom to top) at 160,000g at 4 ^0^C for 4 h in a Beckman L-80 ultracentrifuge using a SW55Ti rotor. Fractions were separated manually from top to bottom and then subjected to immunoblotting. Note that melanosome fraction appears largely at the interfaces between 1.4 M and 2.0 M sucrose densities.

**Semiquantitative PCR and transcript analysis**

RNA from melanocytes was isolated using the RNeasy kit (Qiagen), and the concentration was estimated using a NanoDrop 2000C spectrophotometer (Thermo Scientific). cDNA was prepared using a cDNA synthesis kit (Fermantas). Transcript levels of a gene were analyzed by PCR on a Bio-Rad S1000 Thermal Cycler using gene specific primers (*mRAB9A*: 5’-TCTTGGAGATGGTGGAGTTG and 5’-TCCCAAATCTGCATGGTAAC; *mRAB9B*: 5’-ACCGGCAGAGCTTTGAGAAC and 5’-TACAGCTAACACCTGCCTA; *mRAB38*: 5’- ACGCTCCCTAATGGTAAGCCA and 5’-CTAGGATTTGGCACAGCCAGAGCAGCT; *mRAB32*: 5’- ATGGCGGGCGAGGGACTAGGGCAACA and 5’-TCAAACCATCCAGTGAAGCCA; *mVARP*: 5’-CCATGGCATTGTCCTAGTGC and 5’-AAGGCAAGCAAAACCTGCTC; *mTYRP1*: 5’-CCCCTAGCCTATATCTCCCTTTT and 5’-GCCCTGACAAAGTGGCTCT; *mTYR*: 5’-ATCAGCTCAGTCTATGTCATCCC and 5’-TGCCAAGGCAGAAACCCTGGT; *mSTX13*: 5’-CGTCGGCCTTAAACAATTTCCA and 5’-CAAATGAGACGAGCTGCTCTT; *mGAPDH*: 5’-GAGCCAAACGGGTCATCATCT and 5’- GAGGGGCCATCCACAGTCTT; *GFP*: 5’-CCGCTCGAGATGGTGAGCAAGGGCGAG and 5’-ATAGCGGCCGCTTACTTGTACAGCTCGTCCAT; *GFP-hRAB9A*: 5’- CCGCTCGAGATGGTGAGCAAGGGCGAG and 5’-CATAAGTGAATTCTTCCCAAC) and an equal amount of cDNA from each sample. *GAPDH* was used as a control in the PCR. DNA band intensities were measured using Image Lab 4.1 software. Bands were normalized to *GAPDH*, and the fold change relative to controls was calculated.

**Immunoblotting**

Western blotting of cell lysates was performed using a protocol described previously ([Setty et al., 2007](#_ENREF_8)). γ-Tubulin was used as a loading control in all experiments. Immunoblots were developed with the Clarity Western ECL substrate (Bio-Rad), and the luminescence was captured using Image Lab 4.1 software in a Bio-Rad Molecular Imager ChemiDoc XRS+ imaging system. Protein band intensities were measured using Image Lab 4.1 software. Bands were then normalized to tubulin and the fold change relative to controls was calculated.

**Supplementary References:**

Choudhury, A., Dominguez, M., Puri, V., Sharma, D. K., Narita, K., Wheatley, C. L., Marks, D. L., and Pagano, R. E. (2002). Rab proteins mediate Golgi transport of caveola-internalized glycosphingolipids and correct lipid trafficking in Niemann-Pick C cells. J. Clin. Invest. *109***,** 1541-50.

Ha, L., Ichikawa, T., Anver, M., Dickins, R., Lowe, S., Sharpless, N. E., Krimpenfort, P., Depinho, R. A., Bennett, D. C., Sviderskaya, E. V., et al. (2007). ARF functions as a melanoma tumor suppressor by inducing p53-independent senescence. Proc. Natl. Acad. Sci. USA *104***,** 10968-10973.

Halaban, R., Svedine, S., Cheng, E., Smicun, Y., Aron, R., and Hebert, D. N. (2000). Endoplasmic reticulum retention is a common defect associated with tyrosinase-negative albinism. Proc. Natl. Acad. Sci. USA *97***,** 5889-94.

Morita, S., Kojima, T., and Kitamura, T. (2000). Plat-E: an efficient and stable system for transient packaging of retroviruses. Gene. Ther. *7***,** 1063-6.

Suzuki, T., Li, W., Zhang, Q., Karim, A., Novak, E. K., Sviderskaya, E. V., Hill, S. P., Bennett, D. C., Levin, A. V., Nieuwenhuis, H. K., et al. (2002). Hermansky-Pudlak syndrome is caused by mutations in HPS4, the human homolog of the mouse light-ear gene. Nat. Genet. *30***,** 321-4.

Suzuki, T., Li, W., Zhang, Q., Novak, E. K., Sviderskaya, E. V., Wilson, A., Bennett, D. C., Roe, B. A., Swank, R. T., and Spritz, R. A. (2001). The gene mutated in cocoa mice, carrying a defect of organelle biogenesis, is a homologue of the human Hermansky-Pudlak syndrome-3 gene. Genomics *78***,** 30-7.

Sviderskaya, E. V., Hill, S. P., Evans-Whipp, T. J., Chin, L., Orlow, S. J., Easty, D. J., Cheong, S. C., Beach, D., Depinho, R. A., and Bennett, D. C. (2002). p16(Ink4a) in melanocyte senescence and differentiation. J. Natl. Cancer Inst. *94***,** 446-454.
